# Comprehensive genomic analysis reveals dynamic evolution of endogenous retroviruses that code for retroviral-like protein domains

**DOI:** 10.1186/s13100-020-00224-w

**Published:** 2020-09-17

**Authors:** Mahoko Takahashi Ueda, Kirill Kryukov, Satomi Mitsuhashi, Hiroaki Mitsuhashi, Tadashi Imanishi, So Nakagawa

**Affiliations:** 1 Department of Molecular Life Science, Tokai University School of Medicine, Isehara, Kanagawa 259-1193 Japan; 2 Micro/Nano Technology Center, Tokai University, Hiratsuka, Kanagawa 259-1292 Japan; 3 Present address: Department of Genomic Function and Diversity, Tokyo Medical and Dental University, Bunkyo-ku, Tokyo, 113-8510 Japan; 4 Present address: Department of Genomics and Evolutionary Biology, National Institute of Genetics, Mishima, Shizuoka 411-8540 Japan; 5 Department of Human Genetics, Yokohama City University Graduate School of Medicine, Yokohama, Kanagawa 236-0004 Japan; 6 Department of Genomic Function and Diversity, Tokyo Medical and Dental University, Bunkyo-ku, Tokyo, 113-8510 Japan; 7 Department of Applied Biochemistry, School of Engineering, Tokai University, Hiratsuka, Kanagawa 259-1292 Japan; 8 Institute of Medical Sciences, Tokai University, Isehara, Kanagawa 259-1193 Japan

**Keywords:** Endogenous retrovirus, Retroviral-like protein domain, Open reading frame, Evolution, Divergence pattern, Co-option, de novo gene

## Abstract

**Background:**

Endogenous retroviruses (ERVs) are remnants of ancient retroviral infections of mammalian germline cells. A large proportion of ERVs lose their open reading frames (ORFs), while others retain them and become exapted by the host species. However, it remains unclear what proportion of ERVs possess ORFs (ERV-ORFs), become transcribed, and serve as candidates for co-opted genes.

**Results:**

We investigated characteristics of 176,401 ERV-ORFs containing retroviral-like protein domains (*gag*, *pro*, *pol*, and *env*) in 19 mammalian genomes. The fractions of ERVs possessing ORFs were overall small (~ 0.15%) although they varied depending on domain types as well as species. The observed divergence of ERV-ORF from their consensus sequences showed bimodal distributions, suggesting that a large proportion of ERV-ORFs either recently, or anciently, inserted themselves into mammalian genomes. Alternatively, very few ERVs lacking ORFs were found to exhibit similar divergence patterns. To identify candidates for ERV-derived genes, we estimated the ratio of non-synonymous to synonymous substitution rates (*dN/dS*) for ERV-ORFs in human and non-human mammalian pairs, and found that approximately 42% of the ERV-ORFs showed *dN/dS* < 1. Further, using functional genomics data including transcriptome sequencing, we determined that approximately 9.7% of these selected ERV-ORFs exhibited transcriptional potential.

**Conclusions:**

These results suggest that purifying selection operates on a certain portion of ERV-ORFs, some of which may correspond to uncharacterized functional genes hidden within mammalian genomes. Together, our analyses suggest that more ERV-ORFs may be co-opted in a host-species specific manner than we currently know, which are likely to have contributed to mammalian evolution and diversification.

## Background

Transposable elements (TEs), also known as “jumping genes”, constitute large portions of mammalian genomes. It has been reported that up to 70%, of the human genome originates from TEs [1–3]. Generally, TEs are categorized as junk DNA [4]; however, many studies have shown that they have, in fact, contributed greatly to mammalian evolution [5–7]. Furthermore, TEs promote rearrangement of chromosomal DNA [8], and can become sources of both coding and regulatory sequences during the evolution of host genomes [9–12]. In particular, specific types of long terminal repeat (LTR) retrotransposons, including endogenous retroviruses (ERVs), have been shown to develop the ability to function as genes in several mammalian tissues [13–17]. One of the most well-studied ERV-derived genes is syncytin. Specifically, the human syncytin-1 (*ERVW-1*) gene, which is derived from an envelope (*env*) gene in human endogenous retrovirus (HERV) type W (*HERV-W*)*,* functions in cell fusion during placental development [18–21]. Interestingly, many of the molecular functions of ERV-co-opted genes remain the same as those in viruses [16–18].

ERVs are thought to be derived from retroviruses established in the germ line of various organisms in the past. In the human genome, ERVs occupy approximately 8% of the human genome [1–3]. Many ERVs lose their open reading frames (ORFs) by accumulating deletions or mutations after integration. Thus, it is unclear what proportion of ERVs in mammalian genomes possess retroviral-like protein ORFs, have been under purifying selection, and have maintained high transcriptional potential. Thus far, genome-wide comparative analyses using epigenomic and transcriptomic data have examined the regulatory regions of ERVs [22]; however, they have not characterized ERV-derived protein-coding sequences. Recently, many whole transcriptome sequencing (RNA-Seq) datasets have been accumulated in public databases. One of the most comprehensive RNA-Seq datasets was generated by the genotype-tissue expression (GTEx) study using 31 human tissues [23]. The data was used to assemble a similar pipeline in the Comprehensive Human Expressed SequenceS (CHESS) project and served to generate 20,352 potential protein-coding genes, and 116,156 novel transcripts in the human genome [24]. A comparative analysis study focusing on the protein-coding region of ERVs, especially which are under purifying selection, using the new transcript data, in combination with cap analysis of gene expression (CAGE) data [25], and epigenomic data will provide new insight into the transcriptional potential, and functionality of these regions.

To understand the characteristics of possible protein-coding ERVs that possess ORFs for retroviral-like protein domains (ERV-ORFs), including their transcriptional potential in mammalian genomes, we comprehensively examined ERV-ORFs in 19 mammalian species. We previously developed a database called gEVE (http://geve.med.u-tokai.ac.jp) containing 176,401 ERV-ORFs containing at least one functional viral gene motif found in 19 mammalian genomes [26]. In this study, we analyzed ERV-ORFs in the gEVE database using various transcriptome and epigenomic data in humans and mice including the abovementioned data. Several systematic searches were performed to obtain ERV-ORFs at the domain level; these studies were designed to detect ERVs in the human genome [27–30]. However, most of these studies were limited to protein-coding ERVs in humans or mice, and primarily examined ERV sequences that contained nearly full-length ORFs or specific domains. Importantly, ERV-derived genes have also been described that possess truncated ORFs and play important roles in specific situations [31]. Moreover, multi-exon genes have been identified that contain exons partially derived from ERV sequences [32]. Note that ERV-ORFs stored in the gEVE database do not always possess ORFs starting with an initiation codon (i.e. an ATG triplet); however, all must contain viral-like protein domains that were predicted using Hidden Markov models (HMMs). The identified ORFs were primarily from four ERV genes [*gag* (viral core proteins), *pro* (proteases), *pol* (polymerase), and *env* (envelope)], all of which are commonly found in viral amino acid sequences. In this study, we performed genome-wide comparisons on the number, divergence, genomic distribution, and transcriptional potential for each protein domain in the ERV-ORFs of mammals. Our findings are expected to improve the understanding of the evolution and potential roles of unannotated ERV-derived genes.

## Results

### Characteristics of ERV-ORFs in 19 mammalian genomes

**Fig. 1:**
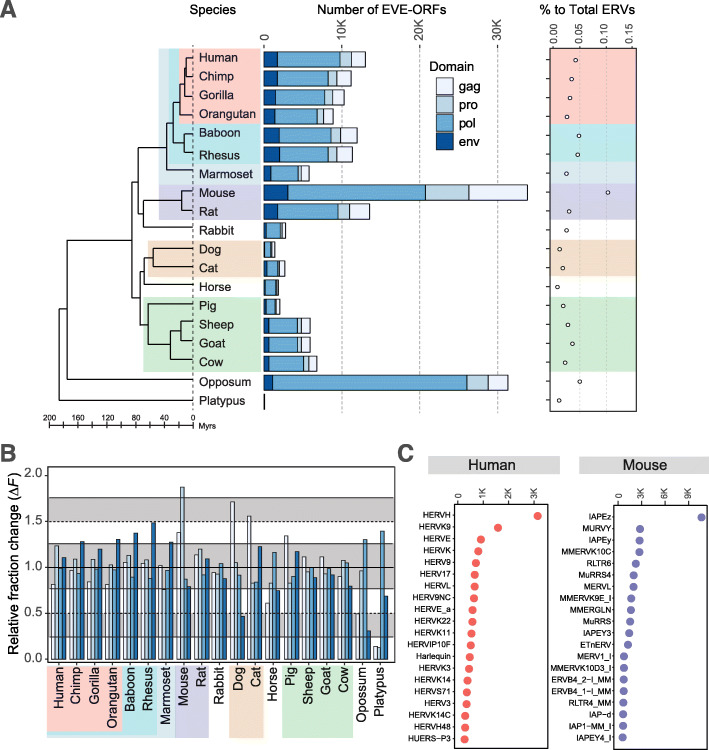
Number of identified ERVs and Repbase annotations in 19 mammalian species. **a** The mammalian phylogeny showing the number of identified ERV-ORFs. Background colors for each species’ name indicate that they have shared species classification equal or below the level of order. Bar colors represent each domain. The proportion of ERV-ORFs identified compared to all ERV sequences in each species, are shown on the right. Chimp, chimpanzee; Rhesus, rhesus macaque monkey. **b** Relative change in the number of each protein domain identified compared to the mammalian average (*∆F*) is shown on the y-axis. Colors of protein domains and mammalian classification are the same as those used in panel **a**. **c** Top 20 Repbase annotations for all ERV-ORFs in the human and mouse genomes. The x-axis represents the number of ERV-ORFs in each annotated group. The ERV categories are significantly enriched in ERV-ORFs when compared to ERVs in each genome (chi-squared test, *p*-value < 0.01, FDR corrected)

To characterize ERVs that are expressed as proteins in mammalian species, we first compared 176,401 possible protein-coding ERV-ORFs from 19 mammalian species genomes obtained from the gEVE database [26]. ERV-ORFs include ORFs of ≥80 amino acid (aa) residues that encode domains in retroviral genes such as *gag*, *pro*, *pol*, and *env* genes. Distribution of ERV-ORF length among mammals are shown in Fig. S1. The number of ERV-ORFs vary among mammalian genomes (Fig. 1a), as we have reported previously [26]. In each genome, the proportion of possible protein-coding ERVs relative to the total ERVs, were found to range from 0.05–0.15% (Fig. 1a). Specifically, within mice and cows, the proportion of detectable ERV-ORFs was higher compared to other species. Alternatively, we identified very few (43) ERV-ORFs in the platypus genome (Fig. 1a); however, this was to be expected as the number of ERVs is very small in this species [33]. We then calculated the proportion of ERV-ORF lengths to those of the total ERVs in the genome of each species, and found that the proportion was correlated (*r* = 0.71, *p* -value < 5.51 × 10^− 4^), which means that the amount of ERV-ORFs in each genome reflected that of the total ERVs in the genome.

We further compared mammalian ERV-ORFs at the level of gene domains. To reduce the influence of genome qualities on the number of domains detected, we calculated the relative fraction change (*∆F*) from the mammalian average for each domain fraction (Fig. 1b). The species-specific characteristics observed in the *∆F* values were found in the *gag* and *env* gene, which exhibited a *∆F* of more than ±0.25 in seven and nine species, respectively (Fig. 1b). The largest difference from the mammalian average in the *env* gene was observed in opossum (*∆F* = 0.3). By contrast, the *∆F* values for *pro* and *pol* were similar among mammalian species, many of which had ∆*F* within ±0.25. Exceptions were found in mice in which the *pro* gene showed a dramatic increase (*∆F* = 1.8). Platypuses showed quite unique compositions in their *gag* and *pro* genes with very small *∆F* values (0.14 and 0.12, respectively).

We next examined which ERV groups from the Repbase database [34] were enriched in human and mice ERV-ORFs (herein we employed the term “group” rather than the Repbase classification of “families/sub-families” for ERVs to avoid confusion with species classification). Top 20 enriched ERV groups were shown in Fig. 1c. A large proportion of the detected ERVs in humans were found to be HERV-H and HERV-K, and IAPE (Intracisternal A-type Particles elements with an Envelope) in mice (Fig. 1c). HERV-H represents both young and ancient HERV groups, which was inserted into the common ancestral genome of simians and prosimians [35], while HERV-K is described as containing younger HERVs [36, 37]. In addition, the Intracisternal A-type Particles elements (IAP) in mice is one of the ERV groups that continue to be active in mice [38]. This high proportion of ERV-ORFs identified in the mouse genome is likely due to the presence of many active ERVs.

**Fig. 2:**
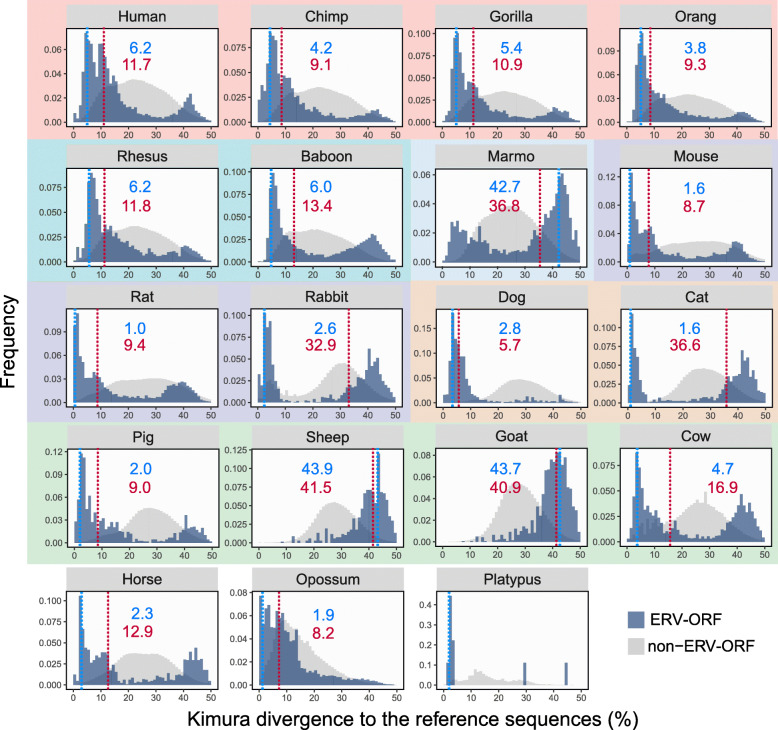
Divergence from Repbase consensus sequences. Divergence frequencies for the consensus sequences in ERV-ORF (blue) and non-ERV-ORF (gray). Background colors for each panel represent the corresponding mammalian classification as indicated in Fig. 1a. For each species, the mode and median values for ERV divergence are shown in blue and red, respectively

We identified divergent fragments of ERV-ORFs using the Repbase consensus sequences and compared them with ERVs excluding ERV-ORF regions (hereafter referred to as non-ERV-ORFs). We hypothesized that newer elements would be closer to the consensus, and older ones would be further. Thus, we were able to estimate the age of ERV-ORFs by comparing the divergence spectra to their consensus sequences. We first determined the medians of Kimura 2-parameter divergence values [39] between ERV-ORFs and Repbase consensus sequences, and found that the medians of divergence were quite different from those of non-ERV-ORFs, which were approximately 22–28% in all eutherians (Fig. 2 and Fig. S2). The ERV-ORFs showed clear bimodal distributions (supported by dip test, *p*-value < 0.01, false discovery rate (FDR) corrected, see Methods) in many mammalian species save for dogs, sheep, goats, and opossum, with two modes of approximately 2–7% (younger) and 39–45% (older). It should be noted that in specific species the distribution patterns revealed that bimodality was not statistically supported in dogs, sheep, goats and opossum (dip test, *p*-value > 0.01, FDR corrected, Fig. 2). Although these distribution patterns were relatively similar among closely related species, such as non-human primates and rodents, overall, the distributions varied significantly between species (Fig. 2).

We next determined how many ERV-ORFs serve as raw materials for the assembly of multi-exon genes. Comparison of our observed ERV-ORFs with those in Ensembl gene and protein annotations revealed that 15 and 22 ERV-ORFs were constituents of ordinary multi-exon genes in humans and mice, respectively (Table S1, non-pink shaded cells for human and mouse genes). It is also intriguing that large numbers of these multi-exon genes were found in sheep (302 out of 324 genes) and opossum (389 out of 396 genes) (Fig. S3). The number of genes containing ERV-ORFs, as predicted by HMM not RepeatMasker [3], were quite large in sheep (18 out of 324 genes) and horse (25 out of 32 genes).

Thus far we observed differences in the presence, type, and divergence of ERV-ORFs among mammals. The high proportion of ERV-ORFs identified in the mouse genome (Fig. 1a) is likely due to the presence of many active ERVs, such as IAP (Figs. 1c and 2). Although some IAP groups contain *env* genes, many do not [40, 41], which may support the observation in mice of relatively higher ∆*F* in *gag* and *pro*, and lower ∆*F* in *env*. These results indicate that many ERV-ORFs in mice may be derived from the young ERVs that possess ORFs by chance. Similar phenomena may have occurred for other species, such as primates, as the frequencies of the divergence with modes of approximately 2–7% were larger than those with 39–45%, which is likely to be boosted by the younger ERVs, such as ERVK (Figs. 1c and 2). However, we also confirmed that the divergence of most known ERV-derived genes in the Ensembl database were also separated into two groups, young and old (Fig. S4), suggesting that not all young ERV-ORFs remained by chance. To identify candidates for ERV-derived genes, it is important to reduce the number of such ERV-ORFs that possess ORFs by chance, which may not function as genes but rather remain ORFs simply because they are too young to have undergone sequence decay. We thus investigated selection pressure as well as transcriptional potential of the ERV-ORFs to better understand the characteristics of ERV-ORFs which are likely to be candidates for ERV-derived genes in mammals.

### Transcriptional potential and selective pressure of ERV-ORF

**Fig. 3:**
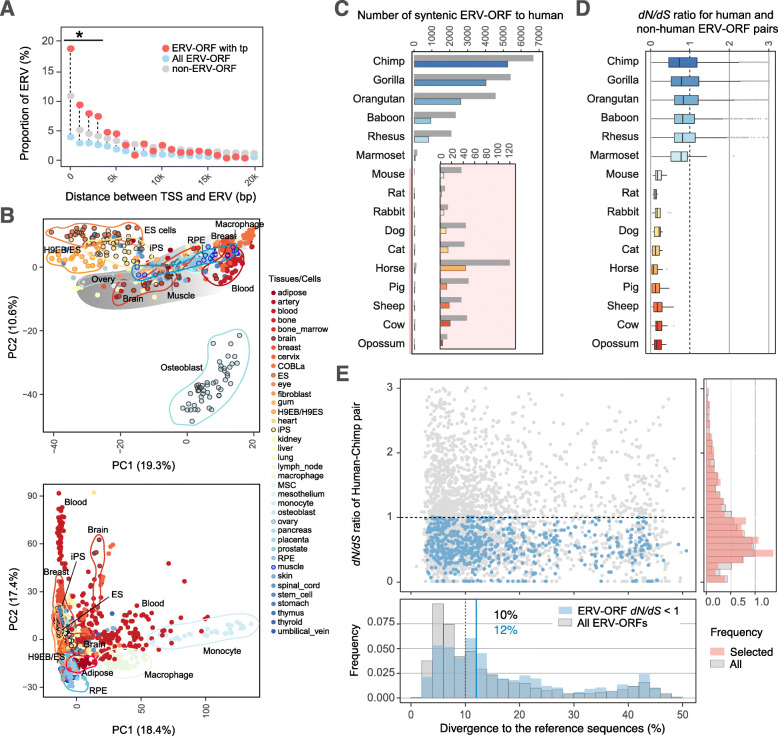
Transcriptional potential and selection of ERV-ORFs. **a** Proportion of ERV-ORFs downstream of TSS as obtained from FANTOM datasets in each 1000 bp bin (all: light blue, transcriptional potential (tp): pink). ERVs without ORFs (non-ERV-ORFs) are shown for comparison (gray). The x-axis represents the distance between ERV-ORFs and the closest TSS. An asterisk (*) indicates statistically significant differences when comparing numbers of the observed ERV-ORF to those expected using proportions of the non-ERV-ORF for each bin (*p* -value < 0.001, chi-squared test, FDR corrected). **b** Top: PCA plot for TSSs from CAGE datasets, located upstream of ERV-ORFs. Colors represent different tissues/cell lines. COBLa_rind, COBL-a (a cell line established from human umbilical cord blood) infected by rinderpest; H9EB/ES, H9 embryoid bodies/embryonic stem cells; MSC, mesenchymal stem cells; RPE, retinal pigment epithelium. Bottom: PCA plot for TSSs from CAGE datasets, located upstream of non-ERV-ORFs. Colors are the same to the panel **c**. **c** The number of ERV-ORFs in mammalian species showing synteny with the human ERV-ORFs. The total number of syntenic ERV-ORFs is shown in gray and non-gray. For the total number of syntenic ERV-ORFs with length > 90% of the human ERV-ORF, each species is shown with a different color. An enlarged graph for the numbers of non-primate species (highlighted with a pink bar on the right side of species names) are shown as an inset. **d** Boxplots of pairwise *dN/dS* ratios for syntenic ERV-ORFs with length > 90% of human ERV-ORFs. Horizontal lines in the middle of each box represent the median value, and edges of boxes are lower and upper quartiles with whiskers as 1.5 times the interquartile range. Single points are beyond the range. **e** Divergence of all ERV-ORFs and ERV-ORFs with transcriptional potential in human-chimpanzee pairs. The scatter plot shows Kimura 2-parameter divergence to Repbase reference sequences (x-axis) and *dN/dS* ratios (y-axis) of ERV-ORFs. ERV-ORFs with transcriptional potential of *dN/dS* < 1, and all ERV-ORFs were shown in blue and gray, respectively. Histograms of divergence and *dN/dS* ratios are shown on the bottom and right of the scatter plot, respectively. For the histogram of divergence, median values of ERV-ORFs with transcriptional potential of *dN/dS* < 1, and all ERV-ORFs are shown in blue and gray, respectively. For the histogram of *dN/dS* ratio, gray and pink represent all ERV-ORFs and ERV-ORFs with transcriptional potential, respectively

To investigate whether ERV-ORFs are expressed as mRNAs, we examined the presence of transcription start sites (TSS) of ERV-ORFs using the CAGE data in the FANTOM5 database [25]. CAGE data are derived from 1816 human samples from various tissues, primary cells, and cell lines. Using this data, we can detect ERV-ORFs with transcriptional potential. We assessed the presence of TSS near all of the ERV data sets, and further compared ERVs with and without ORFs that were located downstream of the TSS. We calculated the number of ERV-ORFs and non-ERV-ORFs located within each bin of 1000 bp from the TSS, and found that significantly fewer ERV-ORFs were located within 20,000 bp of TSS compared to non-ERV-ORFs in humans and mice (Fig. 3a and Fig. S5). We next examined which cell types contain active TSSs located upstream of ERV-ORFs in human expression profiles within CAGE data in the FANTOM5 database. We performed principal component analysis (PCA) analyses using profiles from 38 different cell lines or tissues types, all of which had a minimum of ten available samples for analysis and found that these active TSSs located near ERV-ORFs were segregated into clearly defined groups for different cell types. Principally, in the expression profile of ERV-ORFs, embryonic stem (ES) cells, H9 embryoid body (H9EB) cells, and induced pluripotent stem (iPS) cells were separated into three groups (Fig. 3b, top). Specifically, osteoclasts established a noticeably clear group. Moreover, human body tissue samples, including those from ovaries, muscles, and retinal pigment epithelium (RPE) formed individual groups, part of which overlapped with each other, and merged to form one large group in the profile of ERV-ORFs (Fig. 3b, top). Interestingly, the distinct groups of ERV-ORF differed from those of non-ERV-ORF profiles (Fig. 3b, bottom). Monocytes and macrophages were also clearly separated from iPS, ES, and H9EB/ES cells, suggesting that ERV-ORFs exhibited unique characteristics of transcription that differed from that of non-ERV-ORFs.

However, the ERV-ORFs with transcriptional potential may have been present by chance after recently becoming inserted into the host genome as was observed with an endogenous bornavirus-like nucleoprotein element (EBLNs) expressed in simians [42]. To determine which ERV-ORFs were not simply present by chance, we investigated the type and strength of selective pressure on ERV-ORFs by estimating ratios of non-synonymous to synonymous substitution (*dN/dS*) of ERV-ORFs. To this end, we first identified syntenic sequences to human ERV-ORFs in non-human mammalian genomes, and extracted the sequences when the length of non-human ERV-ORFs was > 90% of that of humans (as shown in Fig. 3c). In this step, we obtained sequence alignments for approximately 58.4% (7524/12,879) of human ERV-ORFs and calculated pairwise *dN/dS* ratios using a maximum likelihood-based approach [43]. The pairwise *dN/dS* ratios of each ERV-ORF were plotted in Fig. 3d and those between human and non-primate mammalian species were under 1, suggesting that, in such diverse species pairs, the ERV-ORFs that conserved the synteny may be under purifying selection (Fig. 3d). Between human and non-human primates, 5414 ERV-ORF pairs showed *dN/dS* < 1; however, the *dN/dS* ratios varied greatly depending on ERV-ORF pairs.

Given that LTR, in particular a 5′ LTR, could potentially function as a promoter for ERV-derived genes, the distance between the gene and their respective TSS should be similar to that of the original ERV structure. Therefore, the distance depends on which ERV domain the ERV-ORFs has originated from. We thus assessed the relationship between TSS and domain types of ERV-ORFs. We first used ERV-ORFs located within ten known ERV-derived single-exon genes (Table S1, pink shaded cells for human and mouse genes) and the closest TSSs in humans and mice; these ERV-ORFs were located approximately 7000 bp from the TSS (Table S1, Dist_TSS column). Four ERV-ORFs in ERV-derived genes were located within 1000 bp of the TSS; however, the remainder, specifically those within the *pol* and *env* domains, were found 1821 bp to 7086 bp downstream of the TSS (Table S1). Within mice, the ERV-ORFs within known ERV-derived genes demonstrated a similar pattern (0 bp to 5402 bp) as that observed in humans (Table S1). The distance range to TSS was relatively long compared to that of ordinary genes, which may have caused by ERV-derived genes using their own 5′ LTR as a promoter. We next applied this result to all our ERV-ORF data sets with *dN/dS* ratios less than 1 in primate pairs, located within 10,000 bp downstream of TSS. We plotted the range of distance between TSS and each domain category of ERV-ORFs with transcriptional potential and found that the median distance increased along the ERV structure of the domain in the order: *gag*-*pro*-*pol*-*env* (Fig. S6A). These results demonstrated that the tendency for TSSs to be located near ERV-ORFs were derived from their own 5′ LTR. However, the distance range was relatively large in all domain categories so that we set a cut-off of 10,000 bp for the distance between TSS and ERV-ORFs to detect ERV-ORFs with transcriptional potential, which can be candidates of ERV-derived genes in this study.

To further understand the relationship between selection pressure and Kimura 2-parameter divergence of ERV-ORFs, we used two groups of ERV-ORFs in the human-chimpanzee pair: the first was located within 10,000 bp downstream of TSS with *dN/dS* ratios less than 1 (ERV-ORFs with transcriptional potential), and the second had no limitation for *dN/dS* ratio or distance to TSS applied (all ERV-ORFs). We extracted 705 ERV-ORFs with transcriptional potential from human-chimpanzee pairs and compared their divergence distributions with those of all ERV-ORFs (Fig. 3e) and found an increase in the median value of the Kimura 2-parameter divergence of ERV-ORFs with transcriptional potential from that of all ERV-ORFs (from 10 to 12%, Wilcoxon rank sum test, *p*-value < 3.1 × 10^− 6^), demonstrating that the divergence of ERV-ORFs with transcriptional potential was significantly higher than that of all ERV-ORFs. Furthermore, the statistical differences in divergence between ERV-ORFs with transcriptional potential and all ERV-ORFs were also observed between human-gorilla and human-orangutan pairs (549 and 428 pairs, Wilcoxon rank sum test, *p*-value < 1.3 × 10^− 6^ and < 8.6 × 10^− 4^, FDR corrected, respectively). This confirmed that ERV-ORFs with relatively small divergence (≤ 7%), which were likely recently inserted into the genomes, may include ERV-ORFs that remain by chance alone. Of note, ERV groups showing a significant decrease in the proportion compared to those expected from all ERV-ORFs in humans were HERV-H and HERV-K groups (Fig. S6B).

### Omics analyses of ERV-ORFs

To predict which ERV-ORFs are expressed in a variety of tissues, we performed comparative analyses using a wide range of transcriptome and histone mark data generated by next-generation sequencing. We first compared observed ERV-ORFs from our study with quantified transcriptome data derived from 31 human tissues generated in the GTEx study [23] from the CHESS database [24]. We found that a total of 279 ERV-ORFs overlapped with transcripts in the CHESS database. Of these, 7 ERV-ORFs were found to correspond to genes containing exons derived from ERV sequences predicted only by HMM, not by RepeatMasker (Table S1). The proportion of ERV-ORFs identified in CHESS transcripts was 3.5%. For each domain, the fraction was 3.0, 2.9, 3.5, and 4.7% in *gag*, *pro*, *pol*, and *env*, respectively. We performed the chi-squared test to examine whether a preference exists for each domain identified as CHESS transcripts. Comparisons of the observed numbers of ERV-ORF domains that were identified as CHESS transcripts with the expected numbers calculated from the fraction of all ERV-ORF domains within the human genome showed that the *env* segment was relatively large compared to the other domains (observed 89, expected 66); however, this result was not statistically significant (chi-square test, *p*-value < 0.16).

**Fig. 4:**
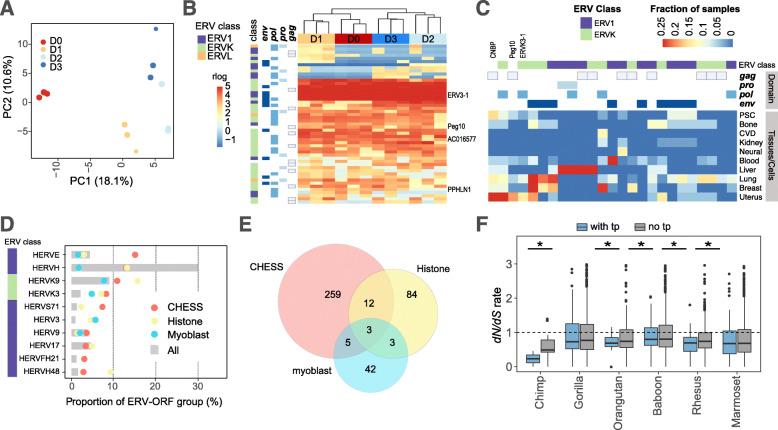
Overlapping human ERV-ORFs with functional data in various tissues and cell lines. **a** PCA plot illustrating the ERV-ORFs expressed during the four stages of human primary myoblast differentiation (three replicates in each stage) for the first two principal components (PC1 and PC2). Differentiation status was indicated by color. D0: undifferentiated myoblasts, D1–3: 1–3 days after myoblast differentiation began. The proportion of contributing variables for a given PC are shown on the axes. **b** Expression levels for ERV-ORFs with ≥10 normalized read counts during human primary myoblast differentiation. ERV classes and domain categories are displayed on the left. The regularized logarithm (rlog) transformed read counts for ERV-ORFs are color-coded from blue (low expression) to red (high expression). Differentiation status is presented in the same color as in panel **a**. **c** Human ERV-ORFs overlapping with H3K36me marks. The fractions of samples of histone marks are shown in heat color scales. ERV classes and domain categories on the top are the same as in the panel **b**. **d** Top 10 ERV groups in ERV-ORFs detected by functional data (chi-squared test, *p* -value < 0.05, FDR corrected). The different ERV sets for all ERV-ORFs in this study (gray bar), detected from the CHESS datasets (pink circle), histone datasets (yellow circle), Myoblast RNA-Seq datasets (blue circle) are indicated. ERV classes on the left are the same in the panel **b**. **e** Overlaps of human ERV-ORFs with transcription potential detected by different functional datasets. Fractions of ERV-ORFs located near TSSs (< 5kbp) in each functional datasets are presented under the name of functional data. **f** Pairwise *dN/dS* ratios for ERV-ORFs with transcriptional potential (tp) and without tp in human are shown in pink and gray, respectively. Dots and error bars represent average rates and standard deviation, respectively

Since myoblast cells were reported to express syncytins during myogenesis [44, 45], we also analyzed RNA-Seq data from human and mouse differentiating myoblasts. The relevant expression data from human primary myoblasts (12 runs) and mouse C2C12 cells (16 runs) were collected from the sequence read archive (SRA) database (see Supplementary Table 3 for details). Following gene mapping and quantification, the expression data of ERV-ORFs having a minimum of ten normalized read counts in the human and mouse myoblasts were extracted. The RNA-Seq data were analyzed from four (Days 0–3) and three (Days 0, 3, 6) time points after differentiation had begun in humans and mice, respectively. PCA plots successfully captured the difference in ERV-ORF expression at each time point (Fig. 4a and Fig. S7). From these plots, 51 and 586 ERV-ORFs were identified as having a minimum of ten normalized read counts in all samples at the same differentiation stage in humans and mice, respectively. In both species, a number of ERV-ORFs exhibited high expression levels throughout the entire differentiation process, including ERV3–1 in humans, and AH000833 and AI506816 in mice (Fig. 4b and Fig. S8). Other known genes/transcripts such as Peg10, AC016577 and PPHLN1 in humans, and syncytin-A and Asprv in mice were also detected, however, their expressions were low. Many ERV-ORFs detected in the myoblast differentiation were unreported transcripts, derived from all four ERV domains of ERV1, ERVK, and ERVL, some of which exhibited stage-specific expression patterns (Fig. 4b and Fig. S8). It is noteworthy to mention that in ERV-ORFs expressed during human muscle cell differentiation, only eight were also identified in GTEx transcripts.

One of the features associated with transcribed gene body is the chromatin mark of trimethylated histone H3 at lysine 36 (H3K36me3) [46, 47]. We, therefore, applied this feature to identify ERV-ORFs with transcriptional potential as proteins. We obtained the histone mark data of H3K36me3 (9 and 6 cell/tissue types in humans and mice, respectively) from ChIP-Atlas [48], and identified the ERV-ORFs overlapping with this mark in a minimum of four samples from each cell/tissue type. From these results we identified 51 and 250 ERV-ORFs overlapping with the mark, in humans and mice, respectively. Further, a higher proportion of samples containing ERV-ORFs with H3K36me3 in each cell/tissue type showed cell type specific patterning (Fig. 4c and Fig. S9). We also identified several ERV-ORFs marked by multiple cell types; some of which were the fourth exon of CNBP (cellular nucleic acid-binding) genes in humans and mice, yet others were unreported ERV-ORFs. All ERV-ORF domains were determined contain the H3K36me3 mark in humans and mice. However, a portion of the domain expression exhibited tissue specificity; for example, in humans, the *pro* domain was found exclusively in liver cells. We also analyzed the relationship between TSSs and ERV-ORFs with transcriptional potential, which were detected by comparing RNA-Seq and histone mark data (Fig. 3a). A significantly larger proportion of ERV-ORFs with transcriptional potential were located downstream of TSS compared to non-ERV-ORFs, especially within 1000 bp in humans [FDR corrected *p*-value < 0.05]. When comparing numbers of the two types of ERV-ORFs within each bin of 2000 bp, we found a significantly larger proportion of ERV-ORFs with transcriptional potential within 4000 bp from TSS compared to non-ERV-ORFs in humans and mice (Fig. 3a and Fig. S5, *p*-value < 0.01, FDR corrected).

We then examined enriched ERV groups based on Repbase classification in ERV-ORFs with transcriptional potential, which were identified using three functional datasets (CHESS, myoblast RNA-Seq, and H3K36me), and compared them with those of all ERV-ORFs detected in the human genome. The top 10 enriched ERV groups in ERV-ORFs with transcriptional potential are shown in Fig. 4d (*p* -value < 0.05, chi-squared test, FDR corrected). Some enriched ERV groups were more prominent in ERV-ORFs with transcriptional potential compared to all ERV-ORFs detected in humans. For example, when comparing top 10 enriched ERV groups, the proportion of the HERVK3 group significantly increased in all three functional datasets compared to that in all ERV-ORFs in humans (Fig. 1c and Fig. S10). By contrast, the proportion of the HERV-H group in ERV-ORFs with transcriptional potential was significantly decreased from that in all ERV-ORFs in humans (Figs. 1c and 4d, and Fig. S10). Similarly, differences in the enriched ERV groups were observed among the three datasets of ERV-ORFs with transcriptional potential. Specifically, high proportions of the HERV-E and HERVS71 groups were observed in ERV-ORFs from the CHESS database, yet were not observed in ERV-ORFs identified using the other functional datasets. Conversely, the fractions of HERVK9 and HERVH48 groups were found to be highly enriched in ERV-ORF from H3K36me3 histone dataset compared to those detected in CHESS datasets. The histone datasets contain a larger variety of samples, including iPS, ES, and cancer cell lines, while CHESS datasets are obtained exclusively from human tissues. In ERV-ORFs from myoblast RNA-Seq, the proportion of the HERV-K group was significantly increased compared to that of all ERV-ORFs detected in humans (Figs. 1c, 4d, chi-squared test, *p* -value < 0.001, FDR corrected), which accounts for nearly 60% of ERV groups in the ERV-ORFs from myoblast RNA-Seq. However, enrichment of the HERV-K group was observed only in ERV-ORFs from myoblast RNA-Seq. Hence, the differences in the ERV-ORF groups may result from differences in sample types used in each project. Indeed, the ERV-ORFs detected via three different functional datasets were quite different (Fig. 4e). Although we identified 408 ERV-ORFs with transcriptional potential, only 15 were identified in both the CHESS and histone datasets. Moreover, since myoblast cells were not included in two of the datasets, only 11 ERV-ORFs were found to be overlapped with those in CHESS and histone datasets. These observations confirmed that ERV-ORFs were tissue-specific, and approximately 3.2% (408/12,879) and 2.3% (752/32,062) of ERV-ORFs may be transcribed as proteins in humans and mice, respectively.

We also compared *dN/dS* ratios between all ERV-ORFs and ERV-ORFs with transcriptional potential in primates, and found that the *dN/dS* ratios with transcriptional potential only for baboons, macaques and marmosets were significantly smaller than those of all ERV-ORFs (*p*-value < 0.05, Wilcoxon rank sum test, FDR corrected). Moreover, the *dN/dS* ratios of ERV-ORFs between apes were significantly small when they were shared by at least four non-human primates (Fig. 4f, Wilcoxon rank sum test, *p*-value < 0.05, FDR corrected). In the sets for chimpanzee, gorilla, and orangutan, approximately 81, 74, and 72% of ERV-ORFs were below *dN/dS* 1, respectively. This showed that ERV-ORFs detected by functional data still contain sequences that are not under purifying selection. Out of 408 ERV-ORFs, which were detected using three different functional datasets, 93 had *dN/dS* < 1, demonstrating that approximately 22.3% of ERV-ORFs considered to exhibit high transcriptional potential, were under purifying selection.

### Genome-wide comparison of ERV-ORFs

**Fig. 5:**
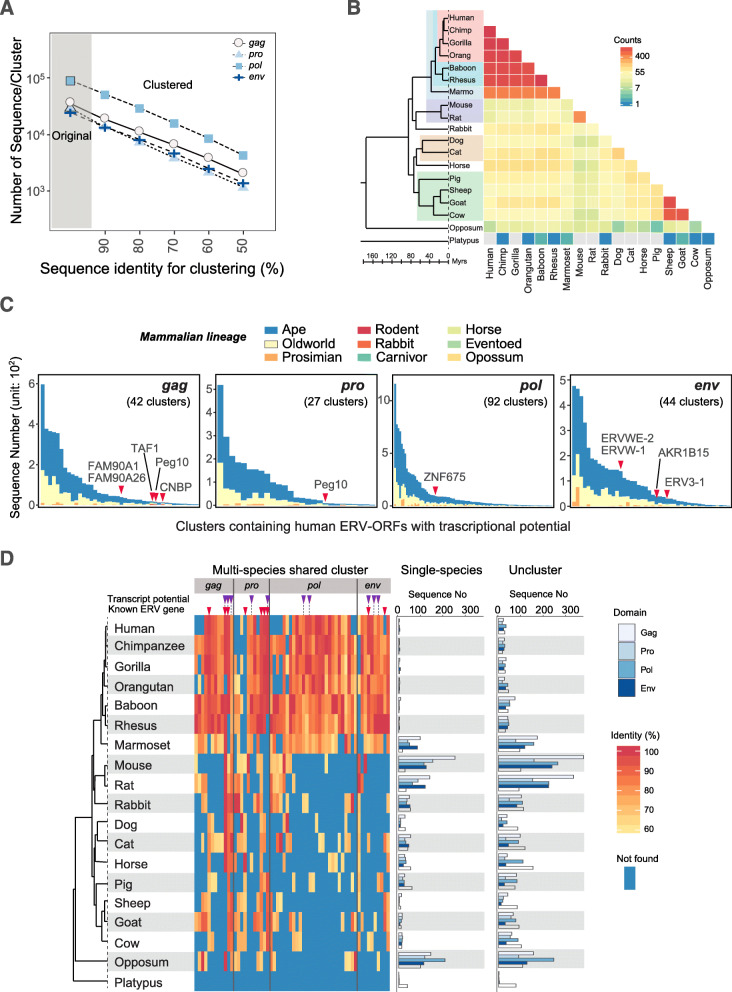
Summary of ERV sequence clustering. **a** Number of ERV-ORF clusters associated with each ERV-ORF domain. The x-axis represents the sequence identity established via clustering. The y-axis represents the number of clusters and also shows the original number of sequences before clustering occurred (gray shaded area) for comparison. A logarithmic scale was used on the y-axis. **b** Pairwise comparison of shared sequences clustered at ≥60% identity level in 19 mammalian species. The color bar represents the number of shared sequences. Gray indicates that no shared sequences were identified. **C)** The number of ERV-ORF sequences in each cluster containing at least one human ERV-ORF identified in the CHESS database. The x-axis represents clusters with ≥60% identity; however, the cluster name is not shown due to limited space. Individual bar colors indicate which ERV-ORFs in each cluster are derived from which species shown in Fig. 1**a** [e.g. Apes (blue) contain ERV-ORFs from human, chimpanzee, gorilla, and/or orangutan]. Clusters containing ERV-derived genes are indicated by red triangles and the specific gene name. **d** ERV-ORF clusters shared among at least eight species at ≥60% identity levels. Domain names are shown on the top. Clusters containing ERV-ORFs with transcriptional potential (purple triangle) and known genes (red triangle) are highlighted. The red-scale color bar represents the percent identities for sequences against a reference sequence within the cluster. The highest identity in each species is shown. Blue represents the absence of a specific sequence in the given cluster. The clusters containing human ERV-ORFs with transcriptional potential (purple triangle) and known mammalian ERV genes (red triangle) were indicated on the top of heatmap. The amount of sequences forming single-species clusters (middle) and those that failed to form any cluster (right) are also shown for each species

The current ERV groups classified by Repbase are based on nucleotide identity; we, therefore, postulated that several unique ERV-derived genes exist that originated from different ERV groups. However, we do not know whether there is certain ERV-ORF domains or amino acid sequences are preferentially retained in different lineages to generate novel ERV-derived genes, including ERV-ORFs, even if they belong to different ERV groups in mammals. We also do not know whether amino acid sequences of ERV-derived genes, including ERV-ORFs, tend to be unique as they exhibit high transcriptional potential. To better understand such relationships between sequence types of ERV-derived genes in mammals, we conducted a genome-wide cluster analysis on ERV-ORFs using CD-HIT [49]. Cross-species ERV clustering provided important predictions for the number of sequence types (different amino acid sequences) in the 19 examined mammalian species. After clustering ERV-ORF sequences, we obtained 96,938 to 8749 clusters at the levels of ≥90% to ≥50% sequence identity, respectively (Fig. 5a). Furthermore, among ERV genes, the number of clusters identified for *pol* genes (Fig. 5a) were more variable, while that for *pro* genes was less variable. We assigned cluster IDs for each cluster by corresponding the number of sequences in the cluster with the length of the reference sequences.

Cluster analysis identified numerous clusters shared between multiple species, which had not been detected by clustering of nucleotide ERV sequences. Generally, genes shared among mammals have ≥60% of shared amino acid identity; however, many ERV-ORFs were shown to be lineage-specific, and made lineage-specific clusters at this identity level; many clusters were found to be shared between primates, rodents, or bovids, whereas less to no, shared clustering occurred in opossum and platypuses (Fig. 5b). This might because opossum and platypus are genetically diverged compared to other mammalian lineages. Using clustering patterns and human RNA-Seq data, we determined the degree of shared ERV-ORFs with transcriptional potential by examining whether the ERV-ORFs were derived from human, primate, or multi-species clusters. Many of the human ERV-ORFs identified in transcripts via RNA-Seq that were detected in transcripts in the CHESS database, and in myoblast differentiation transcriptome data, were also found in clusters of primates (apes and old-world monkeys) at the level of 80% (primate homologs share roughly ≥80% identity). Furthermore, more than half of the clusters contain only small numbers (< 10) of primate ERVs, indicating they are unique sequences (Fig. S11). These human ERV-ORFs with transcriptional potential were also in primate clusters even at the level of ≥60% identity (Fig. 5c). Overall, the clustering of *gag* and *env* domains were shared between similar species; many of which were shared among closely related species such as primates, while other *gag* clusters were shared among distantly related species. Additionally, the *pro* domain cluster, containing transcripts found in the CHESS database, was relatively small, and appeared to be more specific to apes compared to other species gene categories. The clusters containing human ERV-ORFs that overlapped with RNA-Seq transcripts with known ERV gene annotations, are highlighted in Fig. 5c. Many of these genes, save for syncytin-1, were found in clusters comprised of a smaller number of sequences (≤10). Further, of the total 12,879 human ERV-ORFs examined in this study, only 223 sequences were human-specific (un-clustered unique sequences or human-specific clusters). Moreover, from the ERV-ORFs that overlapped with RNA-Seq data transcripts, only two sequences were found to be un-clustered. Mouse ERV-ORFs showed a similar tendency, although the species specificity was stronger than humans. For example, 18,730 sequences out of the total 32,063 ERV-ORFs were mouse-specific, and 349 out of 586 ERV-ORFs detected in the RNA-Seq data were determined to be in mouse-specific clusters. Only 15 ERV-ORFs were unique sequences in the mouse genome.

Many human ERV-ORFs identified via RNA-Seq analysis were found to be shared among primates, while some ERV-ORFs were found in multiple shared clusters. Indeed, of the clusters at the level of 60% identity, we identified 60 clusters that were shared among ≥8 mammalian species (Fig. 5d). The largest shared ERV-ORF domain category was for the *pol* gene, which had 27 clusters shared between species. The remaining ERV gene domains contained a similar number of cross-species clusters (12, 11, and 10 for *gag*, *pro*, and *env*, respectively). Although nine clusters contained known annotated genes or reported transcripts (Fig. 5d and Table S1), many of the ERV-ORFs identified in the clusters were previously unreported; seven clusters contain human ERV-ORFs with transcriptional potential but not known ERV-derived genes. Although no clusters containing mouse ERV-ORFs with transcriptional potential were found, this might be explained by the different number of species for primates and rodents used in the analysis. Interestingly, 15 of these multi-species clusters did not contain human ERV-ORFs.

Nine of these clusters remained following the extraction of ≥8 shared species clusters at the level of ≥80% identity, and 21 clusters remained when extracting clusters shared among ≥10 mammalian species (Fig. S12). Further, there were fewer multi-species ERV-ORF clusters identified in rodents and opossum. Instead, these species exhibited larger numbers of species-specific clusters and unique ERV sequences (Fig. 5d, right panels). The species-specific clusters in these species were abundant in *pol* genes, while there were no multi-species shared clusters observed in analysis of platypuses.

## Discussion

We comprehensively identified ERV-ORFs in 19 mammalian species, with differences in their domain fractions noted between species (Fig. 1a and b). This is the first report, to our knowledge, that presents data on the genome-wide prediction of ERV-ORFs at the domain level in a wide range of mammalian species. We applied *dN/dS* analyses to human ERV-ORFs and found that approximately 42% (5414/12,879) of the ERV-ORFs showed *dN/dS* ratios < 1, which can be considered to be under purifying selection. Further, we confirmed that approximately 9.7% (1243/12,879) of these ERV-ORFs with *dN/dS* < 1 exhibited transcriptional potential. Although the proportion of ERV-ORFs that function as potential candidates for ERV-derived genes was small, there may be unidentified protein-coding genes in these ERV-ORFs.

We also noted that entire ERV-ORFs were significantly depleted near the TSS in humans and mice. This may support the low transcriptional potential of ERV-ORFs as a whole. If specific epigenetic changes occur in close proximity to ERV-ORFs with low transcriptional potential, they subsequently begin to be expressed, which may lead to development of diseases as described in previous studies [50, 51]. This may provide an explanation for why ERV-ORFs become depleted close to TSS in transcriptionally active regions, to avoid potential detrimental secondary effects to their expression. Hence, these preserved ERV-ORFs in transcriptionally inactive regions may function as the raw materials required for constructing new exons for multi-exon genes composed of non-ERV-derived exons during mammalian evolution. Indeed, we identified ERV-ORFs that were exonized in known Ensembl multi-exon genes. Further, the numbers of ERV-containing genes varied among mammalian species (Fig. S3); horse, sheep, and opossum contained a larger number of these genes compared to other mammalian species. It is unclear whether this observation reflected the difference in exonization potential of ERV-ORF or simply in gene annotation quality. Nevertheless, differences, although small, were observed in the number of ERV-ORFs identified in well-annotated human and mouse genomes (Fig. S3). We also found that certain ERV-ORFs partially overlapped with known gene exons (e.g. *AKR1B15, UBXN8,* and *PPHLN1* in humans) containing splice variants. This suggests that, as reported in a previous study [52], ERV-ORFs may be transcribed as alternative transcript variants. It is interesting to note that known multi-exon genes have been identified as containing ERV-ORFs as predicted by HMM alone, suggesting that these ORFs may be originated from ERVs. Moreover, similar numbers of these genes were identified in all examined mammals, save for horses, sheep, and platypuses. Considering that orthologs of these genes were found in different species (e.g. *CNBP, GIN1, SUGP2*, in chickens and *CTSE* in humans and mice), these ERV-ORFs may have been co-opted before the evolutionary divergence of birds and mammals.

The ERV-ORF expression results may also suggest that the promoter regions of ERVs were co-opted rather than the ORFs. Previous studies have reported that promoter regions, most notably for ERV LTRs, have been co-opted as tissue-specific, or alternative promoters in mammalian cells (well summarized in [53]). The LTR promoter usage in a given cell may also be inferred from RNA-Seq data. When analyzing promoter usage, the expression levels were often accumulated and applied to each ERV group, such as HERV-K and MERV-L. Thus, if only a certain ERV-ORF is functional, this accumulated data may cause underestimation of the actual ORF expression levels. In our analysis of myoblast RNA-Seq data, we presented the expression of each ERV-ORF locus separately (Fig. 4b) and confirmed that the level of *dN/dS* ratios in each ERV-ORF loci varied. This showed that ERV-ORFs in the same ERV group experienced different levels of selection including neutral selection. Indeed, we detected 93 human ERV-ORFs showing *dN/dS* < 1 in the comparisons with functional data. We believe this level of detailed expression analysis will assist in revealing the function of ERV-ORFs by identifying individual ERV-ORF loci.

Combinatorial analysis of functional datasets and cluster analysis for ERV-ORFs in humans and mice revealed that many expressed ERV-ORFs were tissue- and lineage-specific. This is consistent with previous studies showing that specific ERV genes, such as *ERVW-1,* are expressed in limited tissues [18, 54–56] and are shared exclusively among apes [57]. In addition, we demonstrated that fractions of each viral-like protein domain in ERV-ORFs varied among mammalian lineages (Fig. 1a and b). Further, ERV-ORF fractions containing transcriptional potential with *dN/dS* < 1 were not significantly different from those of entire ERV-ORFs in humans. Moreover, we observed that the fractions of viral-like protein domains among mammalian linages were quite different, implying that the fractions of retroviral-like domains within ERV-derived genes also differ among mammalian lineages, and are differentially transcribed in different tissues, which may affect lineage-specific characteristics at varying levels.

Together, our results suggest that a certain portion of mammalian ERV-ORFs may be co-opted in a lineage-specific manner, many of which were not described before. Such sequences are likely to have contributed to mammalian evolution and diversification. However, functional data is currently very limited. Thus, further ERV-ORF studies employing the functional data from multiple species may provide a deeper understanding of mammalian evolution.

## Conclusions

We investigated what proportion of ERVs possessing ORFs (ERV-ORFs) are under purifying selection, become transcribed, and serve as candidates for co-opted genes in mammalian genomes using various functional data. We found that approximately 9.7% of the ERV-ORFs showed *dN/dS* < 1, which exhibit high transcriptional potential in humans. We also showed that fractions of each protein domain in the ERVs varied among mammalian species, indicating that the function of ERV-derived genes, which have not been identified yet, may be different among mammals. Taken together, our study suggests that more ERVs may have been co-opted as genes in a lineage-specific manner than we know, which are likely to have contributed to mammalian evolution and diversification.

## Methods

### Extraction of ERV-ORFs

All sequences and annotations for potential protein-coding ERV-ORFs from 19 mammalian genomes were used in this study were obtained from the gEVE database [26] (http://geve.med.u-tokai.ac.jp). Species names and corresponding genome versions used in this study are summarized in Table S2. We classified ERV-ORF sequences by domains predicted by HMMER version 3.1b1 (hmmer.org) with HMM profiles and/or RetroTector [58] as provided in the gEVE database. We further separated ERV *pol* and LINE-ORF2 domains as previously described [26]. We first calculated the fraction of ERV-ORF domains within a species, and obtained the average for each domain across all 19 mammals. We then further divided the original domain fractions for each species by the mammalian average and obtained fold changes for all domains.

### Enrichment of ERV-ORF groups

We first obtained all ERV sequences in the human and mouse genomes using RepeatMasker version 4.03 [3] with the Repbase database (downloaded on Apr 23, 2014) as the reference database. We extracted the ERVs located within ERV-ORFs for each species and calculated the length of ERVs located within the ERV-ORFs by each ERV group, as well as the expected lengths, which were estimated by the proportion of those of all ERVs in the genome. Using the observed and expected length values, we performed chi-square test with chisq.test function implemented in R (*p*-value < 0.001 for both human and mouse). Using adjusted standardized residuals obtained from the test, we further performed the residual analysis. The final *p*-values were calculated by pnorm function of R and adjusted by FDR. For Fig. 4d, expected length values were calculated using all ERV-ORFs, and the expected values were then compared with the observed values of ERV-ORFs detected by three functional datasets (TSS, CHESS, myoblast). To calculate the relative fraction change (*∆F*), we first obtained the total number of each domain (*gag*, *pro*, *pol*, and *env*) and its fraction within a species. Using the fractions, we calculated mammalian average fractions for four domains. Then, each species *∆F* was calculated by dividing the domain fraction of the species by the mammalian average.

### Divergence of ERV-ORFs

We used a utility script “calcDivergenceFromAlign.pl” in RepeatMasker [3] to calculate CpG adjusted divergence information (substitutions levels calculated by Kimura-two parameter [39] for ERV-ORFs). The R package version 3.4.4 [59] was used to calculate the medians for each specie’s genome. We determined the modes of divergence using the R package LaplacesDemon version 16.1.1 [60]. To determine whether the divergence distribution was bimodal, we calculated Hartigans’ dip statistic [61] and *p*-value using the R package diptest [62]. Wilcoxon rank sum tests were performed to compare the differential divergence levels between ERV-ORFs with transcriptional potential and all ERV-ORFs in human-ape pairs with *dN/dS* < 1 using Wilcox.test function of the R package. The *p*-values were adjusted by FDR [63] using the p.adjust function of the R package.

### RNA-Seq analysis

RNA-Seq data for human and mouse myoblast differentiation were downloaded from the NCBI SRA; https:// www.ncbi.nlm.nih.gov/sra). For humans, we used 12 RNA-Seq datasets from skeletal muscle cells under the SRA study number SRP033135 [64]. We obtained 14 RNA-Seq datasets from C2C12 cell differentiation under the SRA number SRP036149 to analyze mouse myoblast differentiation. The accession number of runs used in this analysis are summarized in Table S3. After trimming the sequences using fastp version 0.12.5 [65] with the following set parameters: -q 20 -l 30, reads were mapped onto the human (GRCh38), and mouse (mm10) genomes using HISAT2 version 2.1.0 [66] with the default option. StringTie version 1.3.4b [67] and DESeq2 version 1.18.1 [68] were used to quantify and evaluate EVE expression. In this analysis, we performed mapping and quantification for ERV-ORF transcripts by using in-house ERV gene transfer format (GTF) files, which were available in the gEVE database. After normalizing for size and filtering the data (removing small read counts < 10 aa), we log transformed the count data using the rlog transformation function of the DESeq2, which minimizes the detection of sample differences for transcripts with small counts, and normalizes the counts with respect to the library size. We then performed PCA analysis using the standardized read counts. PCA plots were generated using the built-in R function prcomp. For human RNA-Seq data, expression data was sorted based on the total sum of each ERV-ORF expression profile, and the extracted top 100 ERV-ORF transcripts. The standardized counts were then represented in a heatmap, which was generated using the R package pheatmap version 1.0.10 [69].

### Analysis using Ensembl gene annotations

To investigate whether ERV-ORFs were incorporated in known trassnscript in mammals, we obtained exon coordinates of protein-coding genes from Ensembl Genes 84 database in BioMart. We compared the coordinates of each exon with those of ERV-ORFs using the intersect function of BEDTools version 2.26.0 [70]. We identified overlapping ERV-ORFs with at least 10% of length of overlapping each Ensembl exon.

### Analysis using CHESS

To investigate the functionality of ERV-ORFs, we obtained RNA-Seq datasets from the GTEx project [23] in GFF format from the CHESS v.2.1 database [24]. We generated a bed file from the coordinates of each exon in the GFF file and compared the coordinates with those of ERV-ORFs using the intersect function of BEDTools version 2.26.0 [70]. We then identified ERV-ORFs with at least 10% overlapping exons with CHESS transcripts. For analysis, we used only CHESS transcripts with annotation of protein-coding genes and unknown transcripts.

### Analysis using H3K36me3 histone mark

We obtained histone mark data for H3K36me3 from the Peak Browser in ChIP-atlas [48] with threshold for significance set at 50. To extract mark positions with strong evidence, we used only those marks that were detected in a minimum of five samples. Further, for comparison of H3K36me3 marked ERV-ORFs among cell types, we only used the histone marks obtained from more than ten samples in each tissue or cell.

### Analysis using FANTOM data

To predict which ERV-ORFs become transcribed, we analyzed CAGE data from FANTOM5 [25]. Human and mouse CAGE data (bed files, read count data, and sample information table) were retrieved from the FANTOM5 data repository. We converted ERV-ORF coordinates into hg19 assembly using the UCSC LiftOver tool [71], and identified the ERV-ORF and non-ERV-ORF located downstream of TSSs using the closest function in BEDTools version 2.26.0. To determine which of the differences in number of ERV-ORF and non-ERV-ORFs near TSS were significant, Fisher’s exact test was performed using built-in R function. The expected number of ERV-ORF located downstream of TSSs with a bin size of 1000 bp and 2000 bp was estimated using the fraction of non-ERV-ORF with the same bin size. To control for the FDR, we performed multiple-test correction with FDR [63] using the p.adjust function in the R package. PCA analysis for TSS data was performed using the same procedure as described for the RNA-Seq data. For the PCA plot of TSSs, we used only TSSs located within 2000 bp upstream of ERV-ORFs to reduce the number of data to handle in R.

### *dN/dS* analysis

To predict signals of selections on ERV-ORFs, we generated human-centered pairwise alignments by selecting only non-human sequences with length ≥ 90% of human ERV-ORFs. The nucleotide and amino acid alignments were aligned using MAFFT version 7.394 using the auto option [72]. These aligned sequences were further applied to generate alignments containing the right ORFs by Perl program pal2nal.pl [73]. Using these alignments, we calculated pairwise *dN/dS* ratios with PAML version 4.9i (runmode = − 2, CodonFreq = 2, NSsites = 0) [43]. Wilcoxon rank sum tests were performed to determine statistical significance for *dN/dS* ratios between ERV-ORF groups. The *p*-values were corrected by FDR in the R package.

### Cluster analysis

We obtained ERV-ORF alignments and clustered each domain with the CD-HIT program [49] for 50–90% shared sequence identity. The CD-HIT program clusters sequences by finding the longest reference sequence in each cluster group and performs subsequent calculations to identify all sequences that contain the reference sequence [49]. This clustering step was repeated in a round-robin fashion for each ERV domain category. We assigned cluster IDs for each cluster by corresponding the number of sequences in the cluster with the length of the reference sequences. We then extracted the sequence that contained the highest level of homology with the reference sequence for each species within each cluster and generated matrices for heatmaps. Manipulation of this data was accomplished using in-house Perl scripts. Heatmaps was visualized using the geom_tile function of the R package ggplot2 [74].

### Data manipulations and visualizations

For all analyses employed in this study, we used in-house programs developed with Perl, Python, AWK, and Shell script as well as R package dplyr [75] and rehape [76] for processing and manipulation of the data. For visualization, we used ggplot2 version 2.2.1 with RColorBrewer [77] in the R package.

## Supplementary information

**Additional file 1.**

## Data Availability

The datasets generated and/or analyzed during the current study are available in the gEVE database (http://geve.med.u-tokai.ac.jp), CHESS database (http://ccb.jhu.edu/chess/), FANTOM database (http://fantom.gsc.riken.jp/), SRA database (https://www.ncbi.nlm.nih.gov/sra), and supporting files in the manuscript.

## Abbreviations

ERV: Endogenous retroviruses
ERV-ORF: Endogenous retrovirus open reading frame
tp: Transcriptional potential
TSS: Transcription start site
TE: Transposable element
HMM: Hidden Markov model
CAGE: Cap analysis of gene expression
PCA: Principal component analysis
GTF: Gene transfer format
SRA: Sequence read archive
GTEx: Genotype-tissue expression
CHESS: Comprehensive human expressed sequence
ES: Embryonic stem
EB: Embryoid body
iPS: Induced pluripotent stem

## References

[1] International Human Genome Sequencing Consortium (2001). Initial sequencing and analysis of the human genome. Nature.

[2] de Koning AP, Gu W, Castoe TA, Batzer MA, Pollock DD (2011). Repetitive elements may comprise over two-thirds of the human genome. PLoS Genet.

[3] Smit AFA, Hubley R, Green P. RepeatMasker Open-4.0. 2013–2015. http://www.repeatmasker.org. Accessed 5 July 2019.

[4] Smit AFA (1999). Interspersed repeats and other mementos of transposable elements in mammalian genomes. Curr Opin Genet Dev.

[5] Garcia-Perez JL, Widmann TJ, Adams IR (2016). The impact of transposable elements on mammalian development. Development..

[6] Platt RN, Vandewege MW, Ray DA (2018). Mammalian transposable elements and their impacts on genome evolution. Chromosom Res.

[7] Nishihara H (2019). Transposable elements as genetic accelerators of evolution: contribution to genome size, gene regulatory network rewiring and morphological innovation. Genes Genet Syst.

[8] McVean G (2010). What drives recombination hotspots to repeat DNA in humans?. Phillos Trans R Soc B Biol Sci.

[9] Thornburg BG, Gotea V, Makalowski W (2006). Transposable elements as a significant source of transcription regulating signals. Gene.

[10] Nishihara H, Kobayashi N, Kimura-Yoshida C, Yan K, Bormuth O, Ding Q, Nakanishi A, Sasaki T, Hirakawa M, Sumiyama K (2016). Coordinately co-opted multiple transposable elements constitute an enhancer for wnt5a expression in the mammalian secondary palate. PLoS Genet.

[11] Chuong EB, Elde NC, Feschotte C (2016). Regulatory evolution of innate immunity through co-option of endogenous retroviruses. Science.

[12] Diwash J, Feschotte C, Betran E (2017). Transposable element domestication as an adaptation to evolutionary conflicts. Trends Genet.

[13] Ono R, Nakamura K, Inoue K, Naruse M, Usami T, Wakisaka-Saito N, Hino T, Suzuki-Migishima R, Ogonuki N, Miki H (2006). Deletion of Peg10, an imprinted gene acquired from a retrotransposon, causes early embryonic lethality. Nat Genet.

[14] Matsui T, Miyamoto K, Kubo A, Kawasaki H, Ebihara T, Hata K, Tanahashi S, Ichinose S, Imoto I, Inazawa J (2011). SASPase regulates stratum corneum hydration through profilaggrin-to-filaggrin processing. EMBO Mol Med.

[15] Nakaya Y, Koshi K, Nakagawa S, Hashizume K, Miyazawa T (2013). Fematrin-1 is involved in fetomaternal cell-to-cell fusion in Bovinae placenta and has contributed to diversity of ruminant placentation. J Virol.

[16] Pastuzyn ED, Day CE, Kearns RB, Kyrke-Smith M, Taibi AV, McCormick J, Yoder N, Belnap DM, Erlendsson S, Morado DR (2018). The Neuronal Gene Arc Encodes a Repurposed Retrotransposon Gag Protein that Mediates Intercellular RNA Transfer. Cell.

[17] Ashley J, Cordy B, Lucia D, Fradkin LG, Budnik V, Thomson T (2018). Retrovirus-like gag protein Arc1 binds RNA and traffics across synaptic Boutons. Cell.

[18] Mi S, Lee X, Li X, Veldman GM, Finnerty H, Racie L, LaVallie E, Tang XY, Edouard P, Howes S (2000). Syncytin is a captive retroviral envelope protein involved in human placental morphogenesis. Nature.

[19] Dupressoir A, Lavialle C, Heidmann T (2012). From ancestral infectious retroviruses to bona fide cellular genes: role of the captured syncytins in placentation. Placenta.

[20] Lavialle C, Cornelis G, Dupressoir A, Esnault C, Heidmann O, Vernochet C, Heidmann T (2013). Paleovirology of ‘syncytins’, retroviral env genes exapted for a role in placentation. Philos Trans R Soc Lond B.

[21] Bolze PA, Mommert M, Mallet F (2017). Contribution of syncytins and other endogenous retroviral envelopes to human placenta pathologies. Prog Mol Biol Transl Sci.

[22] Ito J, Sugimoto R, Nakaoka H, Yamada S, Kimura T, Hayano T (2017). Inoue Systematic identification and characterization of regulatory elements derived from human endogenous retroviruses. PLoS Genet.

[23] Carithers LJ, Ardlie K, Barcus M, Branton PA, Britton A, Buia SA, Compton CC, DeLuca DS, Peter-Demchok J, Gelfand ET (2015). A novel approach to high-quality postmortem tissue procurement: the GTEx project. Biopreserv Biobank.

[24] Pertea M, Shumate A, Pertea G, Varabyou A, Breitwieser FP, Chang Y, Madugundu AK, Pandey A, Salzberg SL (2018). CHESS: a new human gene catalog curated from thousands of large-scale RNA sequencing experiments reveals extensive transcriptional noise. Genome Biol.

[25] Lizio M, Harshbarger J, Shimoji H, Severin J, Kasukawa T, Sahin S, Abugessaisa I, Fukuda S, Hori F, Ishikawa-Kato S (2015). Gateways to the FANTOM5 promoter level mammalian expression atlas. Genome Biol.

[26] Nakagawa S, Takahashi MU (2016). gEVE: a genome-based endogenous viral element database provides comprehensive viral protein-coding sequences in mammalian genomes. Database.

[27] Paces J, Pavlícek A, Paces V (2002). HERVd: database of human endogenous retroviruses. Nucleic Acids Res.

[28] de Parseval N, Lazar V, Casella JF, Benit L, Heidmann T (2003). Survey of human genes of retroviral origin: identification and transcriptome of the genes with coding capacity for complete envelope proteins. J Virol.

[29] Villesen P, Aagaard L, Wiuf C, Pedersen FS (2004). Identification of endogenous retroviral reading frames in the human genome. Retrovirology.

[30] Tokuyama M, Kong Y, Song E, Jayewickreme T, Kang I, Iwasaki A (2018). ERVmap analysis reveals genome-wide transcription of human endogenous retroviruses. Proc Natl Acad Sci U S A.

[31] Sugimoto J, Sugimoto M, Bernstein H, Jinno Y, Schust D (2013). A novel human endogenous retroviral protein inhibits cell-cell fusion. Sci Rep.

[32] Sela N, Mersch B, Gal-Mark N, Lev-Maor G, Hotz-Wagenblatt A, Ast G (2007). Comparative analysis of transposed element insertion within human and mouse genomes reveals Alu’s unique role in shaping the human transcriptome. Genome Biol.

[33] Warren WC, Hillier LW, Marshall Graves JA, Birney E, Ponting CP, Grützner F, Belov K, Miller W, Clarke L, Chinwalla AT (2008). Genome analysis of the platypus reveals unique signatures of evolution. Nature.

[34] Bao W, Kojima KK, Kohany O (2015). Repbase update, a database of repetitive elements in eukaryotic genomes. Mob DNA.

[35] Bénit L, Calteau A, Heidmann T (2003). Characterization of the low-copy HERV-fc family: evidence for recent integrations in primates of elements with coding envelope genes. Virology.

[36] Bannert N, Kurth R (2006). The evolutionary dynamics of human endogenous retroviral families. Annu Rev Genomics Hum Genet.

[37] Subramanian R, Wildschutte J, Russo C, Coffin J (2011). Identification, characterization, and comparative genomic distribution of the HERV-K (HML-2) group of human endogenous retroviruses. Retrovirology.

[38] Mouse Genome Sequencing Consortium (2002). Initial sequencing and comparative analysis of the mouse genome. Nature.

[39] Kimura M (1980). A simple method for estimating evolutionary rates of base substitutions through comparative studies of nucleotide sequences. J Mol Evol.

[40] Mietz JA, Grossman Z, Lueders KK, Kuff EL (1987). Nucleotide sequence of a complete mouse intracisternal A-particle genome: relationship to known aspects of particle assembly and function. J Virol.

[41] Dewannieux M, Dupressoir A, Harper F, Pierron G, Heidmann T (2004). Identification of autonomous IAP LTR retrotransposons mobile in mammalian cells. Nat Genet.

[42] Kobayashi Y, Horie M, Tomonaga K, Suzuki Y (2011). No evidence for natural selection on endogenous Borna-like nucleoprotein elements after the divergence of Old World and New World monkeys. PLoS One.

[43] Yang Z (2007). PAML 4: a program package for phylogenetic analysis by maximum likelihood. Mol Biol Evol.

[44] Bjerregard B, Ziomkiewicz I, Schulz A, Larsson LI (2014). Syncytin-1 in differentiating human myoblasts: relationship to caveolin-3 and myogenin. Cell Tissue Res.

[45] Redelsperger F, Raddi N, Bacquin A, Vernochet C, Mariot V, Gache V, Blanchard-Gutton N, Charrin S, Tiret L, Dumonceaux J (2016). Genetic evidence that captured retroviral envelope syncytins contribute to myoblast fusion and muscle sexual dimorphism in mice. PLoS Genet.

[46] Ernst J, Kheradpour P, Mikkelsen TS, Shoresh N, Ward LD, Epstein CB, Zhang X, Wang L, Issner R, Coyne M (2011). Mapping and analysis of chromatin state dynamics in nine human cell types. Nature.

[47] Wagner EJ, Carpenter PB (2012). Understanding the language of Lys36 methylation at histone H3. Nat Rev Mol Cell Biol.

[48] Oki S, Ohta T, Shioi G, Hatanaka H, Ogasawara O, Okuda Y, Kawaji H, Nakaki R, Sese J, Meno C (2018). ChIP-atlas: a data-mining suite powered by full integration of public ChIP-seq data. EMBO Rep.

[49] Li W, Godzik A (2006). Cd-hit: a fast program for clustering and comparing large sets of protein or nucleotide sequences. Bioinformatics.

[50] Perron H, Germi R, Bernard C, Garcia-Montojo M, Deluen C, Farinelli L, Faucard R, Veas F, Stefas I, Fabriek BO (2012). Human endogenous retrovirus type W envelope expression in blood and brain cells provides new insights into multiple sclerosis disease. Mult Scler.

[51] Kassiotis G (2014). Endogenous retroviruses and the development of cancer. J Immunol.

[52] Bae MI, Kim YJ, Lee JR, Jung YD, Kim HS (2013). A new exon derived from a mammalian apparent LTR retrotransposon of the SUPT16H gene. Int J Genomics.

[53] Thompson PJ, Macfarlan TS, Lorincz MC (2016). Long terminal repeats: from parasitic elements to building blocks of the transcriptional regulatory repertoire. Mol Cell.

[54] Muir A, Lever AM, Moffett A (2006). Human endogenous retrovirus-W envelope (syncytin) is expressed in both villous and extravillous trophoblast populations. J Gen Virol.

[55] Søe K, Andersen TL, Hobolt-Pedersen AS, Bjerregaard B, Larsson LI, Delaissé JM. Involvement of human endogenous retroviral syncytin-1 in human osteoclast fusion. Bone 2011; 48:837–846. 10.1016/j.bone.2010.11.011 21111077

[56] Soygur B, Sati L (2016). The role of syncytins in human reproduction and reproductive organ cancers. Reproduction.

[57] Grandi N, Cadeddu M, Blomberg J, Mayer J, Tramontano E (2018). HERV-W group evolutionary history in non-human primates: characterization of ERV-W orthologs in Catarrhini and related ERV groups in Platyrrhini. BMC Evol Biol.

[58] Sperber GO, Airola T, Jern P, Blomberg J (2007). Automated recognition of retroviral sequences in genomic data–RetroTector. Nucleic Acids Res.

[59] R Core Team (2017). R: A language and environment for statistical computing.

[60] Statisticat LLC. LaplacesDemon: Complete Environment for Bayesian Inference. Bayesian-Inference.com. R package version 16.1.1. 2018. https://web.archive.org/web/20150206004624/http://www.bayesian-inference.com/software. Accessed 22 May 2020.

[61] Hartigan JA, Hartigan P (1985). The dip test of unimodality. Ann Stat.

[62] Maechler M (2016). diptest: Hartigan’s Dip Test Statistic for Unimodality -Corrected. R package version 0.75–7.

[63] Benjamini Y, Hochberg Y (1995). Controlling the false discovery rate: a practical and powerful approach to multiple testing. J R Stat Soc Ser A Stat Soc.

[64] Trapnell C, Cacchiarelli D, Grimsby J, Pokharel P, Li S, Morse M, Lennon NJ, Livak KJ, Mikkelsen TS, Rinn JL (2014). The dynamics and regulators of cell fate decisions are revealed by pseudotemporal ordering of single cells. Nat Biotechnol.

[65] Chen S, Zhou Y, Chen Y, Gu J (2018). fastp: an ultra-fast all-in-one FASTQ preprocessor. Bioinformatics.

[66] Kim D, Langmead B, Salzberg SL (2015). HISAT: a fast spliced aligner with low memory requirements. Nat Methods.

[67] Pertea M, Pertea GM, Antonescu CM, Chang TC, Mendell JT, Salzberg SL (2015). StringTie enables improved reconstruction of a transcriptome from RNA-seq reads. Nat Biotechnol.

[68] Love MI, Huber W, Anders S (2014). Moderated estimation of fold change and dispersion for RNA-seq data with DESeq2. Genome Biol.

[69] Kolde R (2015). pheatmap: Pretty Heatmaps. R package version 1.0.8.

[70] Quinlan AR, Hall IM (2010). BEDTools: a flexible suite of utilities for comparing genomic features. Bioinformatics.

[71] Hinrichs AS, Karolchik D, Baertsch R, Barber GP, Bejerano G, Clawson H, Diekhans M, Furey TS, Harte RA, Hsu F (2006). The ucsc genome browser database: update 2006. Nucleic Acids Res.

[72] Katoh K, Standley DM (2013). MAFFT multiple sequence alignment software version 7: improvements in performance and usability. Mol Biol Evol.

[73] Suyama M, Torrents D, Bork P (2006). PAL2NAL: robust conversion of protein sequence alignments into the corresponding codon alignments. Nucleic Acids Res.

[74] Wickham H (2016). ggplot2: elegant graphics for data analysis.

[75] Wickham H, François R, Henry L, Müller K (2018). dplyr: A Grammar of Data Manipulation. R package version 0.7.5.

[76] Wickham H. Reshaping data with the reshape package. J Stat Softw 2007. 21:1–20 http://www.jstatsoft.org/v21/i12/.

[77] Neuwirth E (2014). RColorBrewer: ColorBrewer Palettes. R package version 1.1–2.

