# Supplementary material for: Comprehensive genomic analysis reveals dynamic evolution of endogenous retroviruses that code for retroviral-like protein domains
Source: Mob DNA. 2020 Sep 17;11:29. doi: 10.1186/s13100-020-00224-w (PMC7499964; doi:10.1186/s13100-020-00224-w)
Supplement: Supplementary file 1 — Additional file 1. [file 13100_2020_224_MOESM1_ESM.pdf]

Figure S1 Violin plots of ERV-ORF Length

Frequency distribution of length of all ERV-ORFs **A**) ERV-ORFs, and domains of ERV-ORF **B**) are shown. Average length in each species are indicated by red diamond and letter.

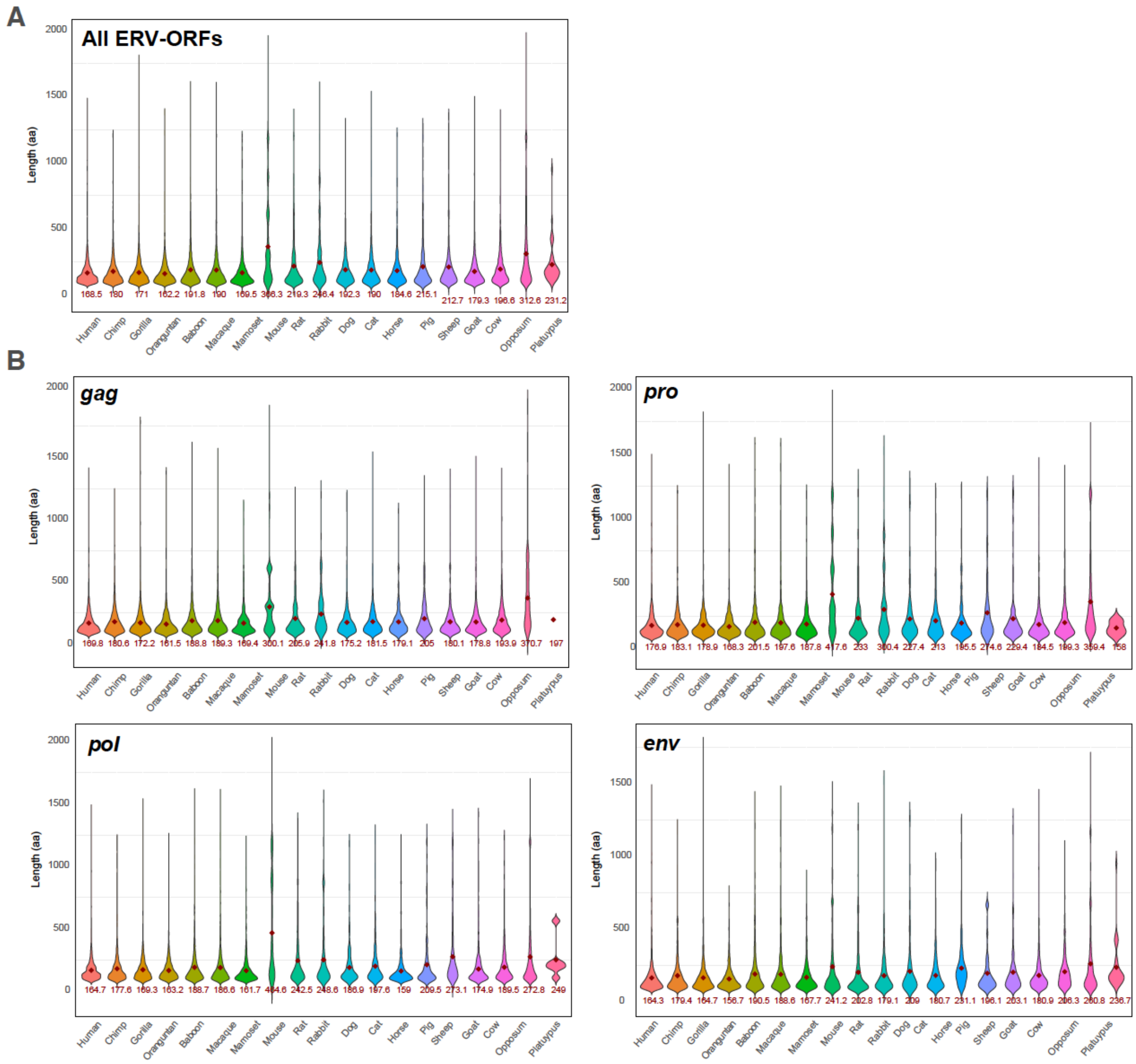

Figure S2 Medians of Kimura divergence for ERV-ORF and non-ERV-ORFs

The plot shows the median values of Kimura divergence for ERV-ORFs (orange), and non-ERV-ORFs (blue). Background colors for each species' name indicate that they have shared species classification equal or below the level of order.

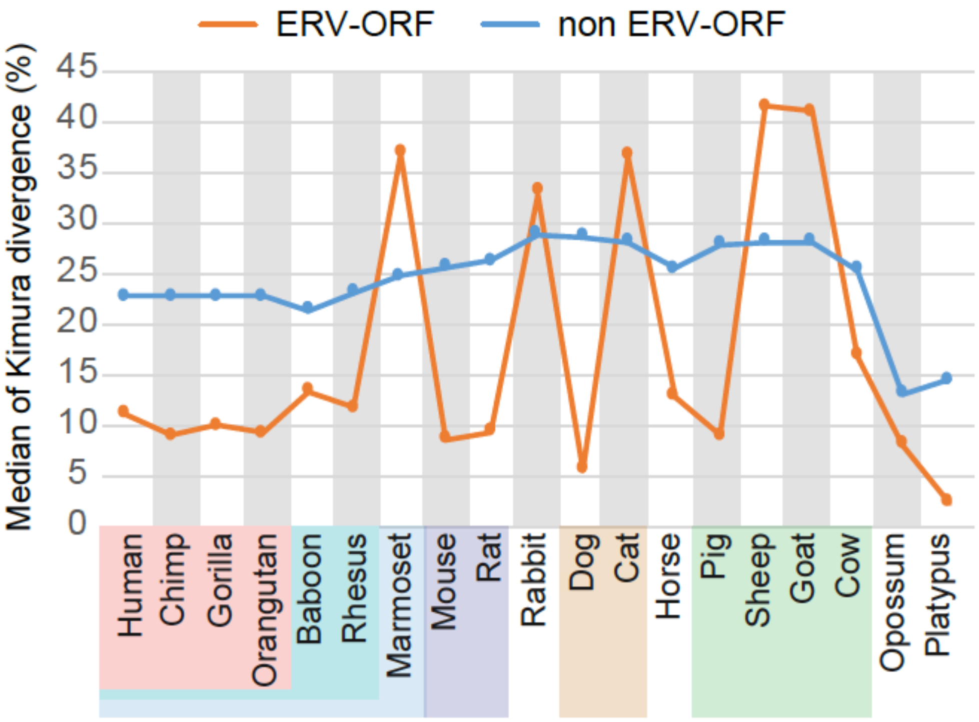

Figure S3 Numbers of Ensembl known genes containing ERV-ORFs

The bar graph shows the number of ERV-derived gene (orange), ERV-ORF containing genes predicted only by HMM (blue), and ERV-ORF containing multi-exon genes (yellow). The number in black on each bar represents the total number of Ensembl genes containing ERV-ORFs. Gene numbers of HMM genes (dark blue) and other multi-exon genes (brown), which are referred in the main text, are shown on the bar.

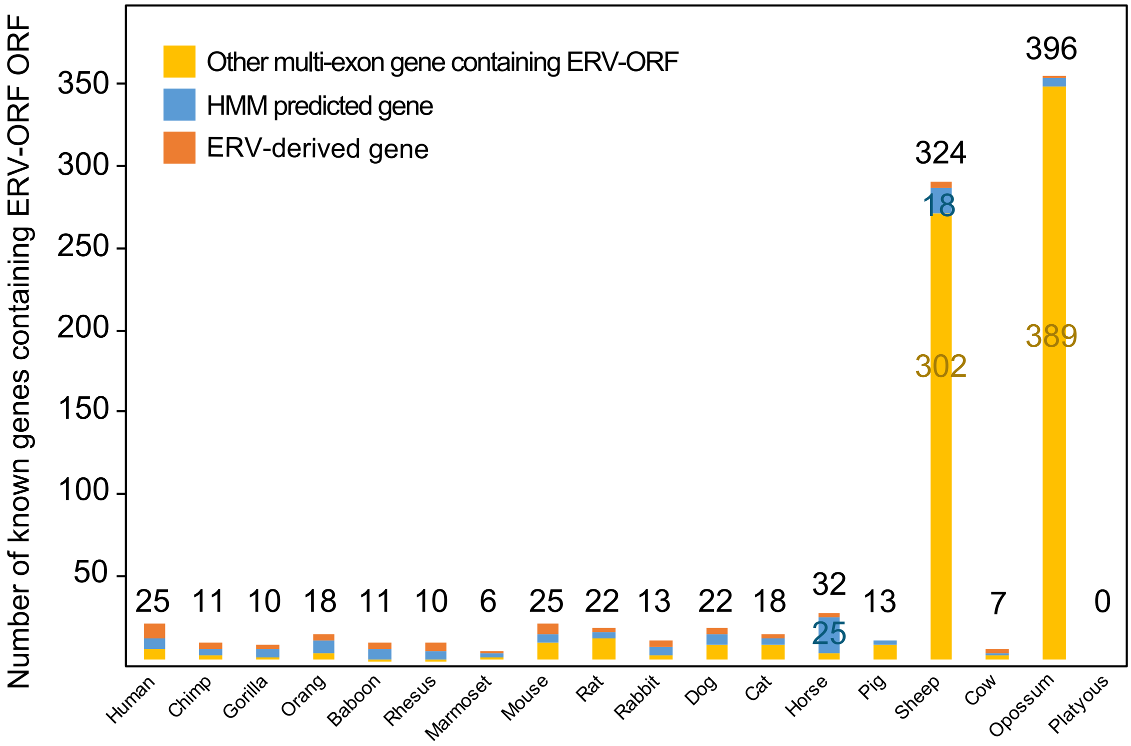

Figure S4 Kimura divergence of known ERV-derived genes containing ERV-ORFs

The bar graph shows the frequency of ERV-ORFs. Background colors for each species' name indicate that they have shared species classification equal or below the level of order. The divergence of known ERV-derived genes containing ERV-ORFs are indicated. Small and large divergences are colored in red and blue.

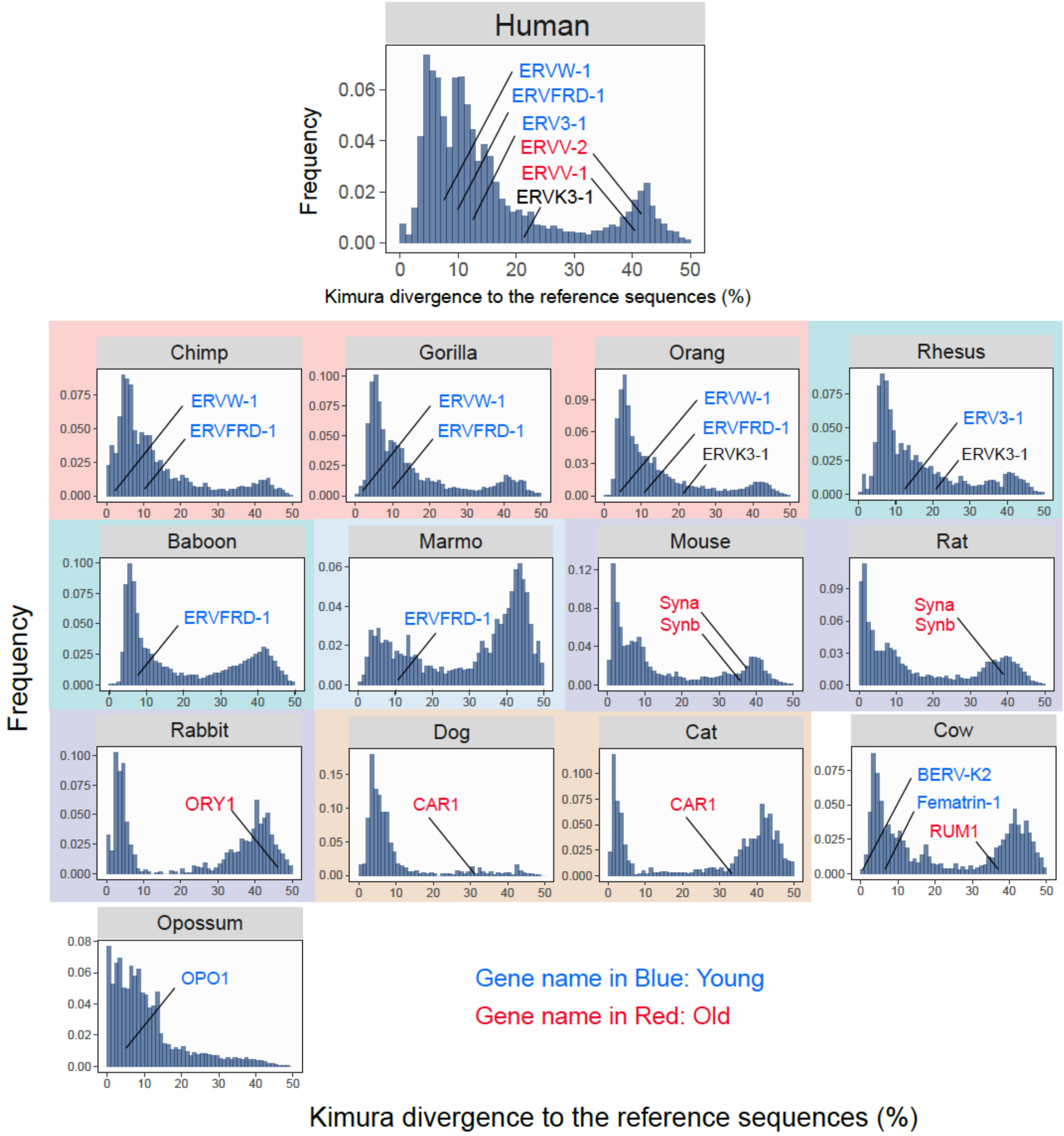

Figure S5 Mouse ERV-ORF vs. non-ERV-ORF

Proportions of three types of ERVs in 1000bp bins according to the distance to TSS. All ERV-ORF, ERV-ORF with detected by functional dataset (ERV-ORF with tp), and ERVs with no ORF (non-ERV-ORF) are shown in light blue, pink, and grey points, respectively. Asterisk (*) indicates statistically significant differences when comparing ERV-ORF with tp and non-ERV-ORF at each distance bin ($p << 0.05$, fisher's exact test, FDR corrected). Comparisons between all ERV-ORFs and non-ERV-ORFs showed statistically significant difference in all bins except for 19k-20kbp.

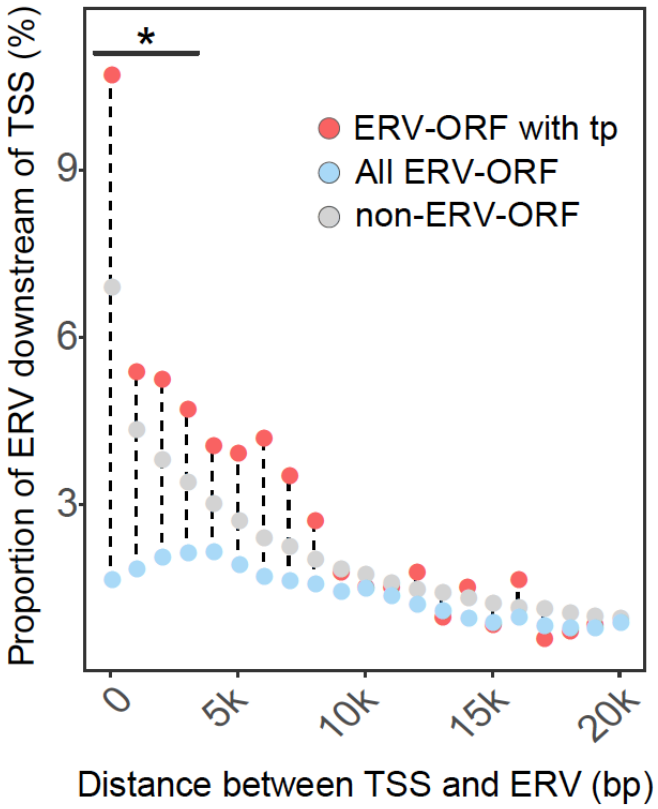

Figure S6 Distance between TSS and ERV-ORFs in humans

A) The distance between TSS and human ERV-ORFs in each domain. Only ERV-ORFs which were located within 10,000bp downstream of TSS showing dN/dS<1 in human and non-human primate pairs (ERV-ORFs with transcriptional potential) were used. B) Top 20 enriched ERV groups of human ERV-ORFs with transcriptional potential (tp). ERV groups in ERV-ORFs with tp showing significant changes in their proportions compared to those in all ERV-ORFs were shown in light blue (residual analysis after chi-squared test, adjusted p-value << 0.001). ERV groups in all ERV-ORFs are indicated by gray.

A

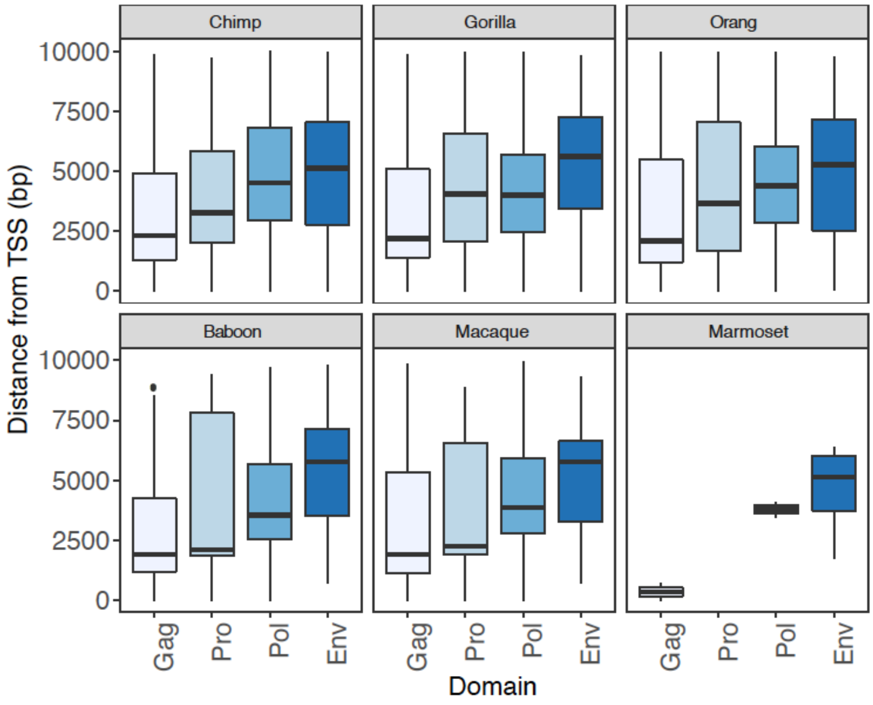

B

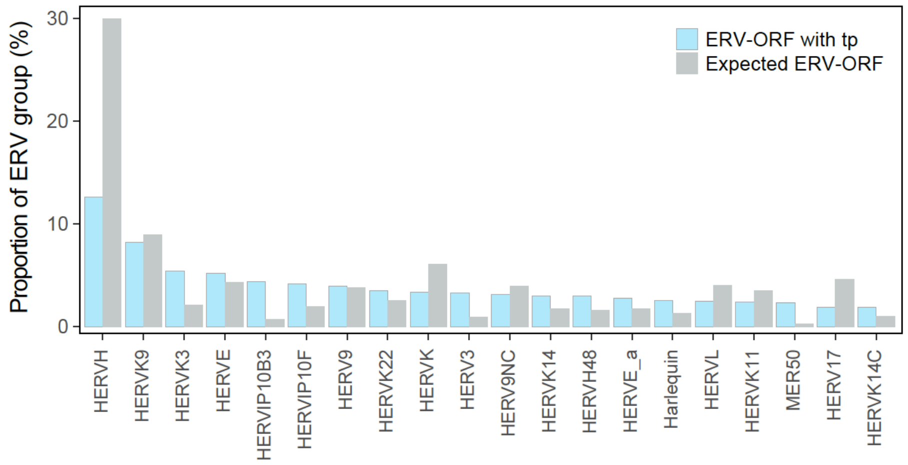

Figure S7 Mouse PCA plot

PCA plot of ERV-ORFs expressed across 3 different stages of myoblast (C2C12) differentiation on the first two principal components (PC1 and PC2). Differentiation status was indicated by color. D0: undifferentiated myoblasts, D3, 6: 3 and 6 days after starting the myoblast differentiation process. The percentage of variation to a given PC is indicated on the axis.

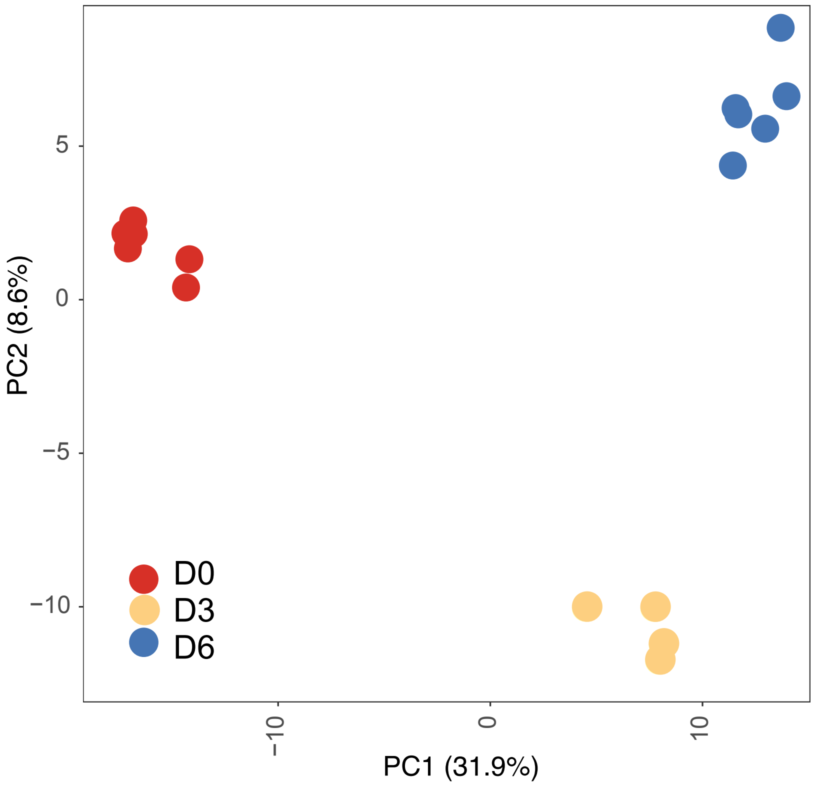

Figure S8 Heatmap of ERV-ORFs in mouse C2C12 cells during differentiation
ERV-ORFs with normalized read counts at least 4 samples of each stage were shown in the heatmap. ERV-ORF types are on the left column. The regularized logarithm (rlog) transformed read counts for ERV-ORF are color coded from blue (low expression) to red (high expression). Differentiation status is shown underneath D0: undifferentiated myoblasts, D3-D6: 3 to 6 days after myoblast differentiation started. The number of ERV-ORFs detected in the RNA-Seq was large so that only ERV-ORFs with read counts ≥50 was shown.

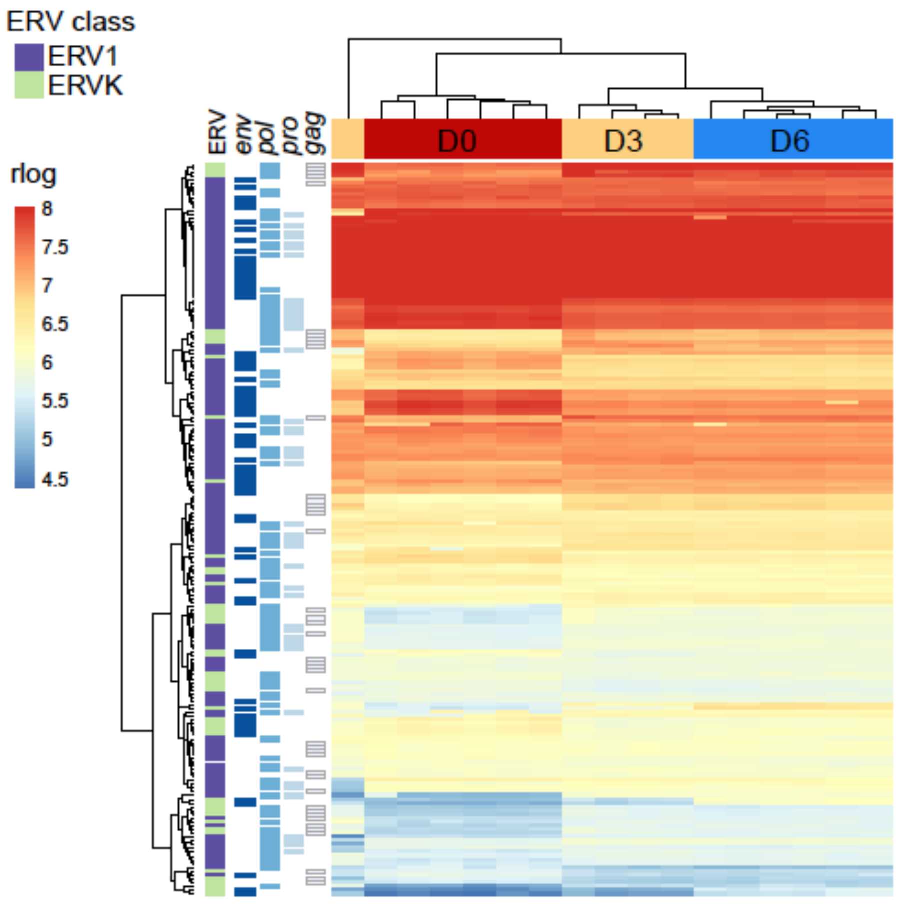

Figure S9 Cell types

ERV-ORFs with ≥10 normalized read counts at least 4 samples were shown in the heatmap. ERV-ORF types are on the left column. The regularized logarithm (rlog) transformed read counts for ERV-ORF are color coded from blue (low expression) to red (high expression). Differentiation status is shown underneath D0: undifferentiated myoblasts, D3-D6: 3 to 6 days after myoblast differentiation started.

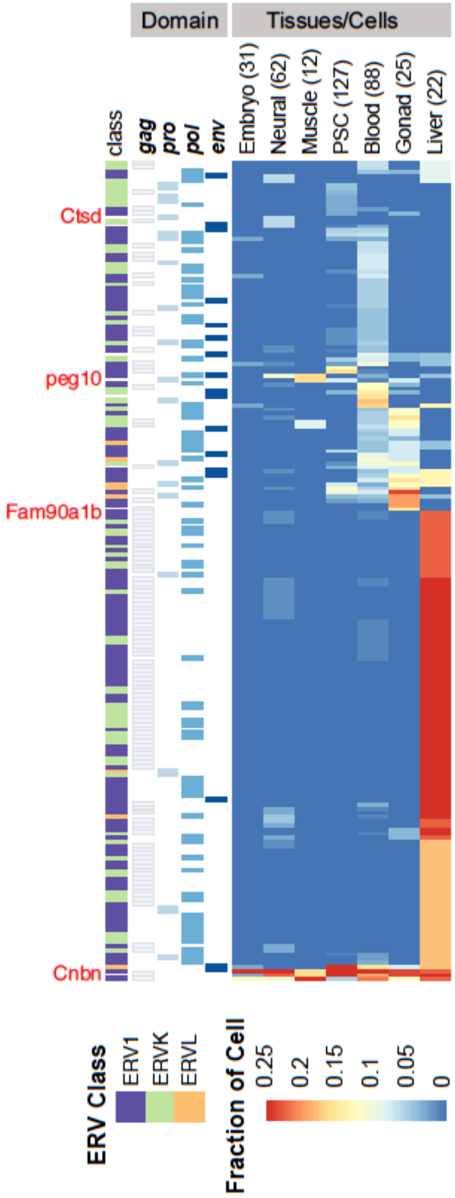

Figure S10 Enriched ERV groups of ERV-ORF with transcriptional potential

Top 10 enriched ERV groups in the human ERV-ORF detected in A) the CHESS transcripts, B) myoblast RNA-seq transcript, and C) H3K36me histone data. ERV-ORFs detected by the functional data and all ERV-ORF were shown in blue and gray, respectively. All ERV groups in the ERV-ORF detected by functional data showed statistically significant changes compared to those in all ERV-ORFs (residual analysis after chi-squared test, adjusted p-value << 0.001).

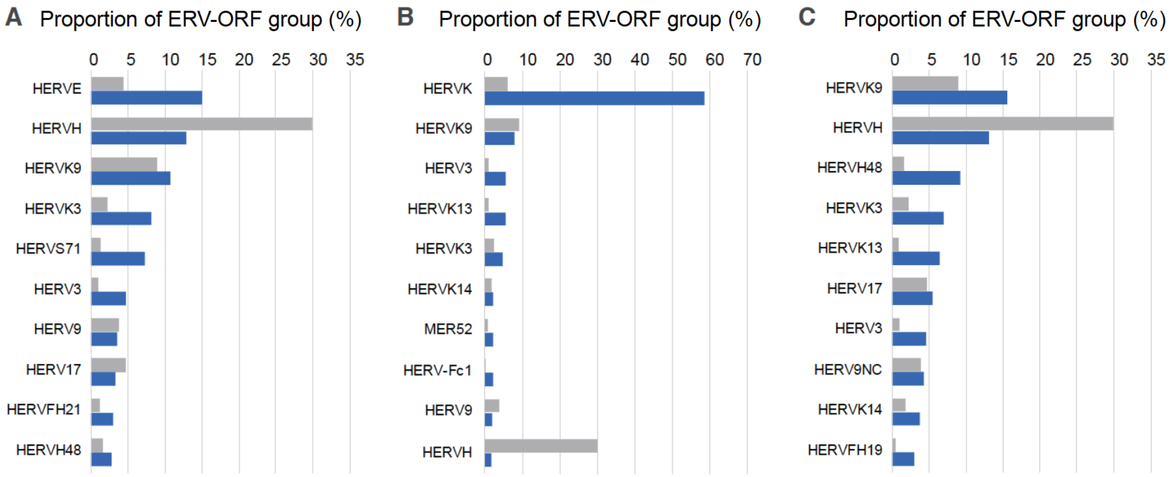

Figure S11 Sequence numbers in each ≥80% level cluster containing ERV-ORFs with transcriptional potential

The number of ERV-ORF sequences in each cluster containing at least one human ERV-ORF with transcriptional potential were shown. The x-axis represents clusters with ≥80% identity. Individual bar colors indicate which ERV-ORFs in each cluster are derived from which species shown in Figure 1A [*e.g*. Apes (blue) contain ERV-ORFs from human, chimpanzee, gorilla, and/or orangutan]. Sequence number at 10 is highlighted by red line. Total cluster numbers in each domain is shown on the top right corner of the panel.

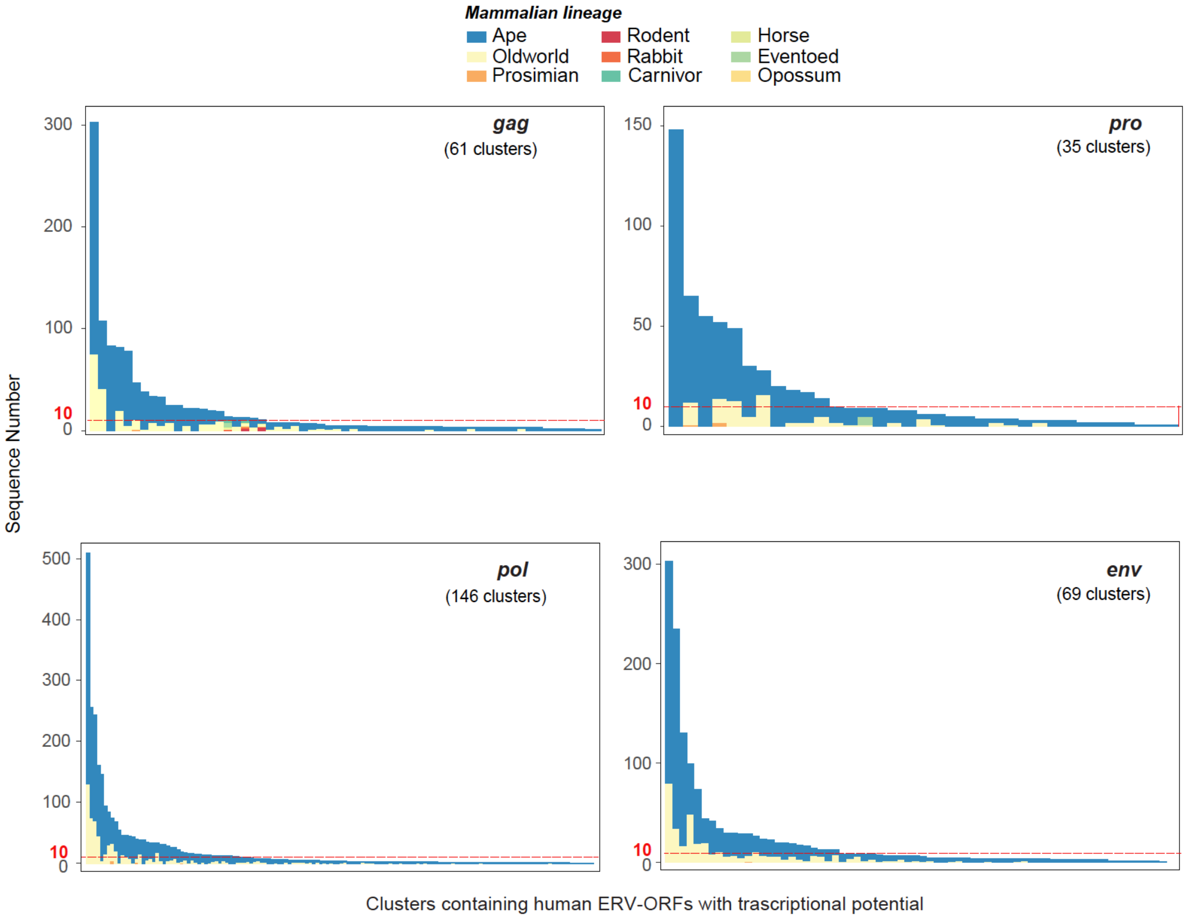

Figure S12 Multi-shared cluster names

ERV-ORFs that are shared among ≥8 species at ≥80% nucleotide identity (A), and among ≥10 species at ≥60% nucleotide identity are shown.

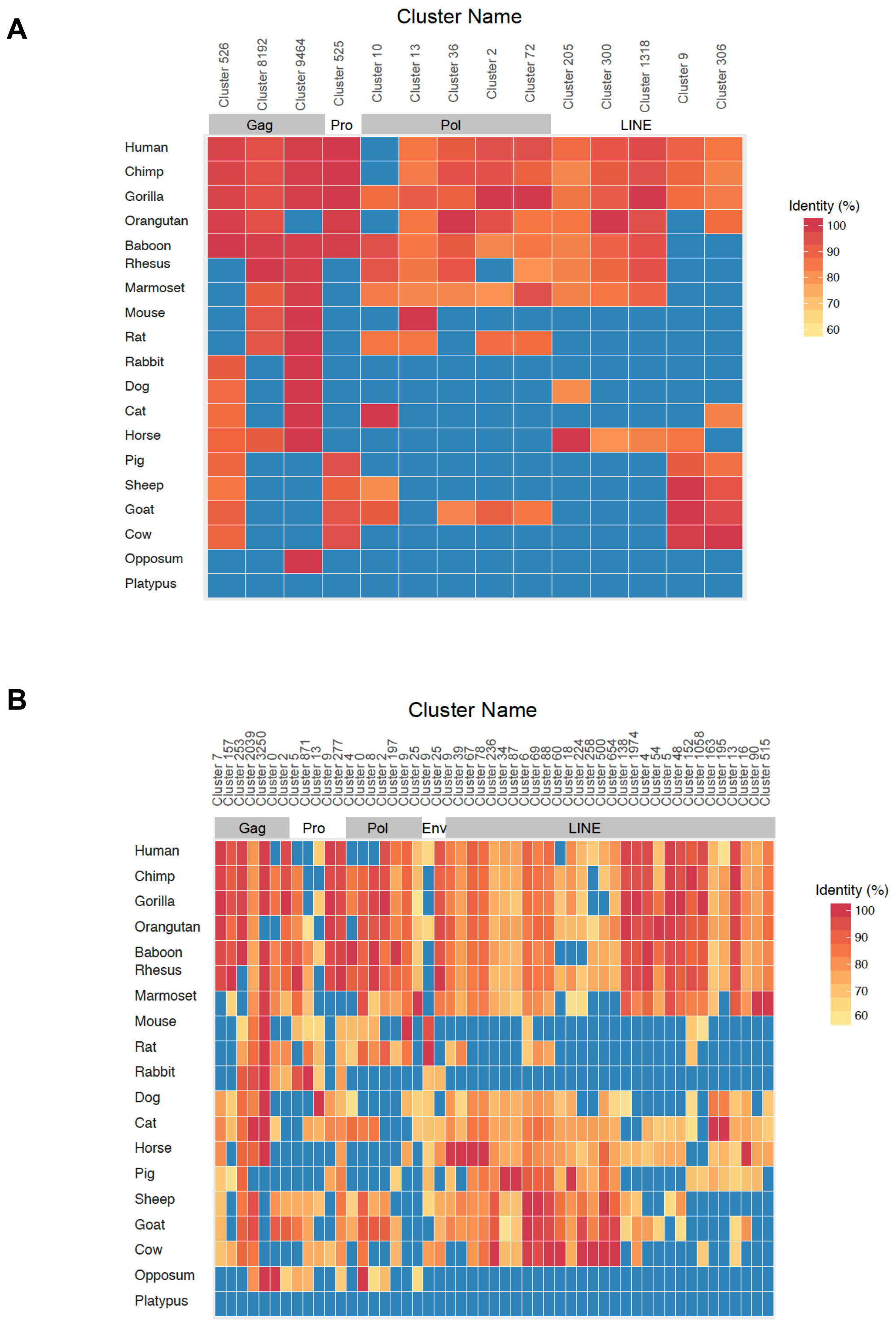

Table S1 ERV-ORFs overlapping with Ensembl annotated gene.

ID: gEVE database ID, **HMM**: viral motives predicted by HMM, **Repbase**: Repbase annotation of ERV-ORFs predicted by RepeatMasker, **Gene**: gene name and Ensembl transcript or gene IDs. We used transcript ID when available. If the transcript has no gene name, “NA” is shown. ERV-ORFs residing within “known ERV-derived single-exon genes“ are indicated by cells colored pink. **Dist_TSS**: the distance to the TSSs is shown only for the ERV-ORFs in the known ERV-derived single-exon genes in humans and mice. The ERV-ORF with only a dot “.” in the Repbase column exhibits the ERV-ORF that was predicted only by HMM.

Human ERV-ORFs

Only human has a column for CHESS annotations (**CHESS**). In the column, ERV-ORFs overlapping status with CHESS transcript is shown; each ERV-ORF has ≥50% overlap, ≥30%, and ≥10% with CHESS transcript, respectively.

<table>
<tr><th>ID</th><th>Domain</th><th>HMM</th><th>Repbase</th><th>Gene</th><th>CHESS</th><th>Dist_TSS</th></tr>
<tr><td>Hsap38.chr2.69960660.69961718.-</td><td>Pro</td><td>Pro:AP;AP_DTG_ILG_template|AP;AP_saspa</td><td>.</td><td>ASPRV1;ENSP00000315383</td><td></td><td>0</td></tr>
<tr><td>Hsap38.chr3.129171078.129171320.-</td><td>Gag</td><td>GAG;GAG_lentiviridae</td><td>.</td><td>PPHP;ENSP00000422110</td><td>≥50%</td><td></td></tr>
<tr><td>Hsap38.chr4.9173574.9173990.+</td><td>Gag</td><td>gag;zf-CCHC_6</td><td>.</td><td>FAM90A26;ENSP00000421131</td><td></td><td></td></tr>
<tr><td>Hsap38.chr5.103087901.103088227.-</td><td>Pro</td><td>INT;GIN1</td><td>.</td><td>GIN1;ENSP00000427162</td><td>≥50%</td><td></td></tr>
<tr><td>Hsap38.chr5.88190260.88190814.-</td><td>Pol</td><td>RNaseH;RNaseH_gammaretroviridae</td><td>HERVE-int;LTR/ERV1|L1MEg;LINE/L1</td><td>TMEM161B;ENSP00000426354</td><td></td><td></td></tr>
<tr><td>Hsap38.chr6.11103697.11105316.-</td><td>Env</td><td>ENV;ENV_deltaretroviridae|ENV;ENV_D-type_betaretroviridae|ENV;ENV_gammaretroviridae|ENV;ENV_retroviridae</td><td>MER50-int;LTR/ERV1</td><td>ERVFRD-1;ENSP00000444461</td><td></td><td>6408</td></tr>
<tr><td>Hsap38.chr7.134564320.134564778.+</td><td>Env</td><td>env;TLV_coat|ENV;ENV_D-type_betaretroviridae|ENV;ENV_gammaretroviridae</td><td>HERVI-int;LTR/ERV1</td><td>AKR1B15;ENSP00000397009</td><td>≥50%</td><td>15405</td></tr>
<tr><td>Hsap38.chr7.64991215.64993059.-</td><td>Env</td><td>ENV;ENV_gammaretroviridae</td><td>HERV3-int;LTR/ERV1</td><td>ERV3-1;ENSP00000391594</td><td></td><td>6410</td></tr>
<tr><td>Hsap38.chr7.92468768.92470387.-</td><td>Env</td><td>env;TLV_coat|ENV;ENV_D-type_betaretroviridae|ENV;ENV_gammaretroviridae</td><td>HERV17-int;LTR/ERV1</td><td>ERVW-1;ENSP00000419945</td><td></td><td>7086</td></tr>
<tr><td>Hsap38.chr7.94663299.94664531.+</td><td>Gag</td><td>GAG;GAG_v_clade</td><td>(CCT)n;Simple_repeat|(CAA)n;Simple_repeat</td><td>PEG10;ENSP00000480676</td><td></td><td>0</td></tr>
<tr><td>Hsap38.chr7.94664474.94665679.+</td><td>Pro</td><td>AP;AP_v_clade</td><td>(ACC)n;Simple_repeat|(CCG)n;Simple_repeat</td><td>PEG10;ENSP00000480676</td><td></td><td>0</td></tr>
<tr><td>Hsap38.chr8.30731656.30732471.+</td><td></td><td>pol;RVT_1|RT;RT_17_6|RT;RT_alpharetroviridae|RT;RT_betaretroviridae|RT;RT_deltaretroviridae|RT;RT_epsilonretroviridae|RT;RT_gammaretroviridae|RT;RT_spumaretroviridae</td><td>HERVE-int;LTR/ERV1</td><td>UBXN8;ENSP00000477532</td><td>≥50%</td><td></td></tr>
<tr><td>Hsap38.chr8.30732333.30732620.+</td><td>Pol</td><td>RT;RT_gammaretroviridae</td><td>HERVE-int;LTR/ERV1</td><td>UBXN8;ENSP00000477532</td><td>≥30%</td><td>4638</td></tr>
<tr><td>Hsap38.chr8.30732669.30733337.+</td><td>Pol</td><td>pol;RNase_H|RNaseH;RNaseH_epsilonretroviridae|RNaseH;RNaseH_gammaretroviridae</td><td>HERVE-int;LTR/ERV1</td><td>UBXN8;ENSP00000483433</td><td>≥50%</td><td>4710</td></tr>
<tr><td>Hsap38.chr9.32631428.32631838.-</td><td>Gag</td><td>gag;zf-CCHC_6</td><td>.</td><td>TAF1L;ENSP00000418379</td><td></td><td></td></tr>
<tr><td>Hsap38.chr12.8224015.8224431.-</td><td>Gag</td><td>gag;zf-CCHC_6</td><td>.</td><td>FAM90A1;ENSP00000445418</td><td>≥50%</td><td>3274</td></tr>
<tr><td>Hsap38.chr12.42444627.42445106.+</td><td>Pol</td><td>pol;RNase_H|RNaseH;RNaseH_betaretroviridae</td><td>HERVK22-int;LTR/ERVK</td><td>PPHLN1;ENSP00000338510</td><td>≥10%</td><td>2306</td></tr>
<tr><td>Hsap38.chr12.42445855.42446199.+</td><td>Pol</td><td>pol;IN_DBD_C|INT;INT_betaretroviridae</td><td>HERVK22-int;LTR/ERVK</td><td>PPHLN1;ENSP00000477681</td><td>≥50%</td><td></td></tr>
<tr><td>Hsap38.chr14.100880715.100884845.-</td><td>Pro</td><td>pro;gag-asp_proteas|AP;AP_v_clade|GAG;GAG_v_clade|RT;RT_17_6|RT;RT_cer2_3|RT;RT_crm|RT;RT_del|RT;RT_gypsy|RT;RT_maggy|RT;RT_pyggy|RT;RT_v_clade</td><td>(GGTG)n;Simple_repeat</td><td>RTL1;ENSP00000435342</td><td></td><td>752</td></tr>
<tr><td>Hsap38.chr19.18995047.18995343.-</td><td>Pro</td><td>pro;G-patch</td><td>.</td><td>SUGP2;ENSP00000470915</td><td>≥50%</td><td></td></tr>
<tr><td>Hsap38.chr19.53013986.53015521.+</td><td>Env</td><td>env;TLV_coat|ENV;ENV_D-</td><td>HERV4_I-int;LTR/ERV1</td><td>ERVV-1;ENSP00000473153</td><td></td><td>1821</td></tr>
<tr><td>Hsap38.chr19.53049147.53050856.+</td><td>Env</td><td>env;TLV_coat|ENV;ENV_D-</td><td>MER66-int;LTR/ERV1</td><td>ERVV-2;ENSP00000472919</td><td></td><td>4506</td></tr>
<tr><td>Hsap38.chr19.58311871.58312203.+</td><td>Pol</td><td>pol;IN_DBD_C|INT;INT_alpharetroviridae|INT;I</td><td>HERVK3-int;LTR/ERVK</td><td>ERVK3-1;ENSP00000489088</td><td></td><td>6243</td></tr>
<tr><td>Hsap38.chr19.23574222.23574482.-</td><td>Pol</td><td>INT;INT_betaretroviridae</td><td>HERVK9-int;LTR/ERVK</td><td>ZNF675;ENSP00000473217</td><td></td><td></td></tr>
<tr><td>Hsap38.chrX.71401441.71401740.+</td><td>Gag</td><td>gag;zf-CCHC_6</td><td>MER8;DNA/TcMar-Tigger</td><td>TAF1;ENSP00000406549</td><td>≥50%</td><td></td></tr>
</table>

Chimpanzee ERV-ORFs

ID	Domain	HMM	Repbase	Gene
Ptro214.chr3.132592082.132592324.-	Gag	GAG;GAG_lentiviridae	.	CNBP;ENSPTRP00000026477
Ptro214.chr5.103437732.103438058.-	Pol	INT;GIN1	.	GIN1;ENSPTRP00000054536
Ptro214.chr5.27254075.27254353.+	Pol	INT;INT_epsilonretroviridae|INT;INT_gammaretroviridae	HERVE-int;LTR/ERV1	TMEM161B;ENSPTRP00000029207
Ptro214.chr6.11189490.11191109.-	Env	ENV;ENV_deltaretroviridae|ENV;ENV_D-type_betaretroviridae|ENV;ENV_gammaretroviridae|ENV;ENV_retroviridae	MER50-int;LTR/ERV1	ERVFRD-1;ENSPTRP00000058789
Ptro214.chr7.92995230.92996849.-	Env	env;TLV_coat|ENV;ENV_D-type betaretroviridae| ENV; ENV_gammaretroviridae	HERV17-int;LTR/ERV1	ERVW-1
Ptro214.chr7.136045679.136046137.+	Env	env;TLV_coat|ENV;ENV_D-type_betaretroviridae|ENV;ENV_gammaretroviridae	HERVI-int;LTR/ERV1	AKR1B15;ENSPTRP00000058399
Ptro214.chr7.95191982.95193214.+	Gag	GAG;GAG_v_clade	(CCT)n;Simple_repeat|(CAA)n;Simple_repeat	PEG10;ENSPTRP00000045828
Ptro214.chr7.95193157.95194362.+	Pro;G-patch	AP;AP_v_clade	(ACC)n;Simple_repeat|(CCG)n;Simple_repeat	PEG10;ENSPTRP00000045828
Ptro214.chr8.51811074.51811343.+	Pol	RT;RT_alpharetroviridae|RT;RT_betaretroviridae	AluYGibC1;SINE/Alu|HERVK9-int;LTR/ERVK	RPL9;ENSPTRP00000061373
Ptro214.chr12.8478239.8478652.-	Gag	gag;zf-CCHC_6	.	FAM90A1;ENSPTRP00000050473
Ptro214.chr19.19312408.19312704.-	Pro	pro;G-patch	.	SUGP2;ENSPTRP00000045722

Gorilla ERV-ORFs

ID	Domain	HMM	Repbase	Gene
Ggor31.chr1.197650039.197650293.-	Pro	pro;G-patch	.	GPATCH2;ENSGGOP00000025170
Ggor31.chr2a.71259560.71260618.-	Pro	AP;AP_DTG_ILG_template|AP;AP_saspase	.	ASPRV1;ENSGGOP00000024375
Ggor31.chr3.129088032.129088274.-	Gag	GAG;GAG_lentiviridae	.	CNBP;ENSGGOP00000009839
Ggor31.chr5.85945229.85945555.-	Pol	INT;GIN1	.	GIN1;ENSGGOP00000026207
Ggor31.chr6.11566308.11567927.-	Env	ENV;ENV_deltaretroviridae|ENV;ENV_D-type_betaretroviridae|ENV;ENV_retroviridae	MER50-int;LTR/ERV1	ERVFRD-1;ENSGGOP00000021598
Ggor31.chr7.89729672.89731291.-	Env	env;TLV_coat|ENV;ENV_D-type betaretroviridae| ENV; ENV_gammaretroviridae	HERV17-int;LTR/ERV1	ERVW-1
Ggor31.chr7.103145635.103146048.-	Pol	RNaseH;RNaseH_betaretroviridae	(CGG)n;Simple_repeat|HERVK9-int;LTR/ERVK	YBX1;ENSGGOP00000017832
Ggor31.chr12.40175362.40175706.+	Pol	pol;IN_DBD_C|INT;INT_betaretroviridae	(GCAGCCCCC)n;Simple_repeat|HERVK22-int;LTR/ERVK	PPHLN1;ENSGGOP00000017687
Ggor31.chr19.19372895.19373191.-	Pro	pro;G-patch	.	SUGP2;ENSGGOP00000021642
Ggor31.chrX.68663891.68664187.+	Gag	gag;zf-CCHC_6	MER8;DNA/TcMar-Tigger	TAF1;ENSGGOP00000015303

Orangtan ERV-ORFs

ID	Domain	HMM	Repbase	Gene
Pabe2.chr1.32354186.32354437.+	Pro	pro;G-patch	.	GPATCH2;ENSPPYG00000000216
Pabe2.chr2a.40839531.40840589.+	Pro	AP;AP_DTG_ILG_template|AP;AP_saspase	.	ASPRV1;ENSPPYG00000012330
Pabe2.chr3.108279426.108280022.-	Pol	INT;INT_alpharetroviridae|INT;INT_betaretroviridae|INT;INT_lentiviridae	HERVK9-int;LTR/ERVK	XYLB;ENSPPYG00000014007
Pabe2.chr3.7275585.7275947.-	LINE	pro;dUTPase	L1MB1;LINE/L1	NA;ENSPPYG00000013463
Pabe2.chr5.103510614.103510940.-	Pol	INT;GIN1	.	GIN1;ENSPPYG00000015666
Pabe2.chr6.11476609.11478228.-	Env	ENV;ENV_deltaretroviridae|ENV;ENV_D-type_betaretroviridae|ENV;ENV_retroviridae	MER50-int;LTR/ERV1	ERVFRD-1;ENSPPYG00000016231
Pabe2.chr7.81421560.81422765.-	Pro	AP;AP_v_clade	(GGTGGC)n;Simple_repeat|(GTG)n;Simple_repeat	NA;ENSPPYG00000029380
Pabe2.chr7.83678858.83680477.+	Env	env;TLV_coat|ENV;ENV_D-type betaretroviridae| ENV; ENV_gammaretroviridae	HERV17-int;LTR/ERV1	ERVW-1
Pabe2.chr8.7379820.7380095.-	Gag	gag;zf-CCHC_6	.	NA;ENSPPYG00000018331
Pabe2.chr9.29027142.29027552.+	Gag	gag;zf-CCHC_6	.	TAF1L;ENSPPYG00000019145
Pabe2.chr11.61285274.61285540.+	Pro	AP;AP_nix1	.	NRIP3;ENSPPYG00000003499
Pabe2.chr12.41813010.41813336.+	Pol	pol;IN_DBD_C	HERVK22-int;LTR/ERVK	PPHLN1;ENSPPYG00000004423
Pabe2.chr14.102426095.102430228.-	Pro:Gag	pro;gag-asp_proteas|AP;AP_v_clade|GAG;GAG_v_clade|RT;RT_17_6|RT;RT_cer2_3|RT;RT_crm|RT;RT_del|RT;RT_gypsy|RT;RT_maggy|RT;RT_pyggy|RT;RT_v_clade	(GGTG)n;Simple_repeat	RTL1;ENSPPYG00000006149
Pabe2.chr17.20152211.20152498.-	Pol	pol;RNase_H	.	NA;ENSPPYG00000008067
Pabe2.chr19.54996417.54996668.-	Gag	gag;zf-CCHC_6	.	NA;ENSPPYG00000010352
Pabe2.chr19.60435534.60435866.+	Pol	pol;IN_DBD_C|INT;INT_alpharetroviridae|INT;INT_betaretroviridae	HERVK3-int;LTR/ERVK	ERVK3-1;ENSPPYG00000029674
Pabe2.chr21.44310326.44310838.-	Env	(env)	.	NA;ENSPPYG00000011460
Pabe2.chr21.44310894.44311181.-	Pol	INT;INT_gammaretroviridae	HERVH48-int;LTR/ERV1	NA;ENSPPYG00000011460
Pabe2.chrX.68901666.68901965.+	Gag	gag;zf-CCHC_6	MER8;DNA/TcMar-Tigger	TAF1;ENSPPYG00000020453

Baboon ERV-ORFs

ID	Domain	HMM	Repbase	Gene
Panu2.chr3.107351109.107352314.−	Pro	AP;AP_v_clade	(GGC)n;Simple_repeat|(GTG)n;Simple_repeat	PEG10;ENSPANP00000010940
Panu2.chr3.107352257.107353555.−	Gag	GAG;GAG_v_clade	(GTT)n;Simple_repeat	PEG10;ENSPANP00000010940
Panu2.chr4.11017359.11018975.−	Env	ENV;ENV_gammaretroviridae|ENV;ENV_retroviridae	MER50-int;LTR/ERV1	ERVFRD-1;ENSPANP00000010950
Panu2.chr6.96802166.96802492.−	Pol	INT;GIN1	.	GIN1;ENSPANP00000011998
Panu2.chr7.157054388.157058521.−	Pro:Gag	pro;gag-asp_proteas|AP;AP_v_clade|GAG;GAG_v_clade|RT;RT_17_6|RT;RT_cer2_3|RT;RT_crm|RT;RT_del|RT;RT_gypsy|RT;RT_maggy|RT;RT_pyggy|RT;RT_pyret|RT;RT_v_clade	.	RTL1;ENSPANP00000000445
Panu2.chr11.83922569.83922811.+	Gag	GAG;GAG_lentiviridae	.	CNBP;ENSPANP00000003453
Panu2.chr12.36760606.36761160.+	Pro	pro;dUTPase|DUT;DUT_caulimoviruses|DUT;DUT_lentiviridae	.	NA;ENSPANP00000009281
Panu2.chr13.68869338.68870462.−	Pro	AP;AP_DTG_ILG_template|AP;AP_saspase	.	ASPRV1;ENSPANP00000007998
Panu2.chr14.1438165.1438500.−	Pro	pro;Asp|AP;AP_pepsins_A1a	.	NA;ENSPANP00000007098
Panu2.chr19.17555770.17556051.−	Pro	pro;G-patch	.	SUGP2;ENSPANP00000000126
Panu2.chrX.62805510.62805842.+	Gag	gag;zf-CCHC_6	MER8;DNA/TcMar-Tigger	TAF1;ENSPANP00000008327

Rhesus magaque ERV-ORFs

ID	Domain	HMM	Repbase	Gene
Mmul1.chr11.85671618.85671860.+	Gag	GAG;GAG_lentiviridae	.	CNBP;ENSMMUP00000015215
Mmul1.chr2.8788584.8789783.+	LINE	pol;RVT_1	L1_RS1;LINE/L1	NA;ENSMMUP00000040168
Mmul1.chr3.60406566.60408392.+	Env	ENV;ENV_gammaretroviridae	HERV3-int;LTR/ERV1	ERV3-1;ENSMMUP00000035333
Mmul1.chr4.110696699.11071315.−	Env	ENV;ENV_deltaretroviridae|ENV;ENV_D-type_betaretroviridae|ENV;ENV_retroviridae	MER50-int;LTR/ERV1	ERVFRD-1
Mmul1.chr5.128640286.128640867.+	LINE	pol;RVT_1	L1-2_Cja;LINE/L1	NA;ENSMMUP00000030240
Mmul1.chr6.154841863.154843983.+	LINE	pol;RVT_1	L1_RS1;LINE/L1	NA;ENSMMUP00000033787
Mmul1.chr6.99336613.99336939.−	Pol	INT;GIN1	.	GIN1;ENSMMUP00000010230
Mmul1.chr7.164164659.164168792.−	Pro:Gag	pro;gag-asp_proteas|AP;AP_v_clade|GAG;GAG_v_clade|RT;RT_17_6|RT;RT_cer2_3|RT;RT_crm|RT;RT_del|RT;RT_gypsy|RT;RT_maggy|RT;RT_pyggy|RT;RT_pyret|RT;RT_v_clade	.	RTL1;ENSMMUP00000007234
Mmul1.chr13.70221349.70222476.−	Pro	AP;AP_DTG_ILG_template|AP;AP_saspase	.	ASPRV1;ENSMMUP00000011804
Mmul1.chr14.1669280.1669618.−	Pro	pro;Asp|AP;AP_pepsins_A1a	.	NA;ENSMMUP00000005169
Mmul1.chr19.59382968.59383237.+	Gag	gag;zf-CCHC_6	.	NA;ENSMMUP00000021136
Mmul1.chr19.64064515.64065216.+	Pol	INT;INT_betaretroviridae	AluYRb3;SINE/Alu|HERVK3-int;LTR/ERVK	ERVK3-1;ENSMMUP00000027005
Mmul1.chrX.123292284.123293480.+	LINE	pol;RVT_1	L1_RS1;LINE/L1	NA;ENSMMUP00000040834
Mmul1.chrX.70302480.70302812.+	Gag	gag;zf-CCHC_6	MER8;DNA/TcMar-Tigger	TAF1;ENSMMUP00000007484

Marmoset ERV-ORFs

ID	Domain	HMM	Repbase	Gene
Cjac321.chr4.17531888.17533507.+	Env	ENV;ENV_D-type betaretrovidae |ENV;ENV_retroviridae	MER50-int;LTR/ERV1	ERVFRD-1:ENSCJAP00000040973
Cjac321.chr2.104319490.104319867.+	Pol	INT;GIN1	.	GIN1;ENSCJAP00000016966
Cjac321.chr6.70290164.70292944.−	LINE	pol;RVT_1	L1-1_Cja;LINE/L1|Sat-1_TSy;Satellite	LYPD6B;ENSCJAP00000005582
Cjac321.chr13.6531633.6531890.+	Gag	gag;zf-CCHC_6	.	NA;ENSCJAP00000007145
Cjac321.chr15.67817302.67817544.+	Gag	GAG;GAG_lentiviridae	.	CNBP;ENSCJAP00000032138
Cjac321.chr22.17949261.17949596.−	Pro	pro;G-patch	.	SUGP2;ENSCJAP00000036644
Cjac321.chrX.63332816.63333115.+	Gag	gag;zf-CCHC_6	MER8;DNA/TcMar-Tigger	NA;ENSCJAP00000043164

Mouse ERV-ORFs

<table>
<tr><th>ID</th><th>Domain</th><th>HMM</th><th>Repbase</th><th>Gene</th><th>Status</th><th>Dist_TSS</th></tr>
<tr><td>Mmus38.chr1.131672160.131672417.+</td><td>Pro</td><td>pro;Asp|AP;AP_pepsins_A1a</td><td>.</td><td>Ctse;ENSMUSP00000108030</td><td></td><td>0</td></tr>
<tr><td>Mmus38.chr1.173568891.173569334.–</td><td>Pol</td><td>INT;INT_betaretroviridae</td><td>ERVB2_1-I_MM;LTR/ERVK</td><td>Pydc4;ENSMUSP00000117222</td><td></td><td></td></tr>
<tr><td>Mmus38.chr1.173695398.173695781.+</td><td>Pol</td><td>INT;INT_betaretroviridae</td><td>ERVB2_1-I_MM;LTR/ERVK</td><td>Pydc3;ENSMUSP00000128958</td><td></td><td></td></tr>
<tr><td>Mmus38.chr1.78497642.78498025.–</td><td>Env</td><td>env;TLV_coat|ENV;ENV_D-type_betaretroviridae|ENV;ENV_gammaretroviridae</td><td>MMERGLN-int;LTR/ERV1</td><td>BC035947;ENSMUSP00000132488</td><td></td><td></td></tr>
<tr><td>Mmus38.chr1.78497809.78500529.–</td><td>Pol</td><td>pol;rve|env;TLV_coat|ENV;ENV_deltaretroviridae|ENV;ENV_D-type_betaretroviridae|ENV;ENV_gammaretroviridae|ENV;ENV_retroviridae|INT;INT_athila|INT;INT_b_clade|INT;INT_csrn1|INT;INT_deltaretroviridae|INT;INT_epsilonretroviridae|INT;INT_gammaretroviridae|INT;INT_gmr1|INT;INT_lentiviridae|INT;INT_osvaldo|INT;INT_spumaretroviridae|INT;INT_tat</td><td>MMERGLN-int;LTR/ERV1</td><td>BC035947;ENSMUSP00000132488</td><td></td><td></td></tr>
<tr><td>Mmus38.chr1.97792275.97792568.+</td><td>Pol</td><td>INT;GIN1</td><td>.</td><td>Gin1;ENSMUSP00000027571</td><td></td><td></td></tr>
<tr><td>Mmus38.chr2.155021472.155024216.+</td><td>Pol</td><td>pol;IN_DBD_C|pol;Integrase_Zn|pol;RNase_H|pol;rve|pol;RVT_1|pol;RVT_thumb|INT;INT_alpharetroviridae|INT;INT_b_clade|INT;INT_betaretroviridae|INT;INT_deltaretroviridae|INT;INT_gammaretroviridae|INT;INT_lentiviridae|INT;INT_spumaretroviridae|RNaseH;RNaseH_betaretroviridae|RT;RT_alpharetroviridae|RT;RT_betaretroviridae|RT;RT_del|RT;RT_deltaretroviridae|RT;RT_epsilonretroviridae|RT;RT_lentiviridae|RT;RT_spumaretroviridae</td><td>ERVB4_1-I_MM;LTR/ERVK</td><td>Gm14226;ENSMUSP00000122157</td><td></td><td>2603</td></tr>
<tr><td>Mmus38.chr2.155024056.155026182.+</td><td>Env</td><td>env;TLV_coat|ENV;ENV_deltaretroviridae|ENV;ENV_D-type_betaretroviridae|ENV;ENV_gammaretroviridae|ENV;ENV_retroviridae</td><td>ERVB4_1-I_MM;LTR/ERVK</td><td>Gm14226;ENSMUSP00000122157</td><td></td><td>4968</td></tr>
<tr><td>Mmus38.chr3.137672404.137675022.+</td><td>LINE</td><td>pol;RVT_1</td><td>L1Md_A;LINE/L1</td><td>Gm21962;ENSMUSP00000126626</td><td></td><td></td></tr>
<tr><td>Mmus38.chr4.150904482.150905285.–</td><td>Pro</td><td>pro;dUTPase</td><td>MERVL-int;LTR/ERVL</td><td>Park7;ENSMUSP00000122265</td><td></td><td>2215</td></tr>
<tr><td>Mmus38.chr5.134558243.134560099.–</td><td>Env</td><td>ENV;ENV_gammaretroviridae|ENV;ENV_retroviridae</td><td>RodERV21-int;LTR/ERV1</td><td>Syna;ENSMUSP00000116437</td><td>SINGL</td><td>3742</td></tr>
<tr><td>Mmus38.chr5.23701149.23703218.–</td><td>Env</td><td>env;TLV_coat|ENV;ENV_deltaretroviridae|ENV;ENV_D-type_betaretroviridae|ENV;ENV_gammaretroviridae|ENV;ENV_retroviridae</td><td>MuLV-int;KTR/ERV1</td><td>Fv4;NA</td><td>SINGL</td><td>0</td></tr>
<tr><td>Mmus38.chr6.129859271.129863149.+</td><td>LINE</td><td>pol;RVT_1</td><td>L1Md_T;LINE/L1|L1Md_Gf;LINE/L1</td><td>Gm17631;ENSMUSP00000130863</td><td></td><td></td></tr>
<tr><td>Mmus38.chr6.4754122.4755348.+</td><td>Gag</td><td>GAG;GAG_v_clade</td><td>(CAA)n;Simple_repeat|(GAT)n;Simple_repeat</td><td>Peg10;ENSMUSP00000127306</td><td></td><td>0</td></tr>
<tr><td>Mmus38.chr6.4755117.4757237.+</td><td>Pro</td><td>AP;AP_v_clade</td><td>(ATCTGC)n;Simple_repeat|(CCA)n;Simple_repeat</td><td>Peg10;ENSMUSP00000127306</td><td></td><td>0</td></tr>
<tr><td>Mmus38.chr6.86628075.86629190.+</td><td>Pro</td><td>AP;AP_DTG_ILG_template|AP;AP_saspase</td><td>.</td><td>Asprv1;ENSMUSP00000046121</td><td>SINGL</td><td>0</td></tr>
<tr><td>Mmus38.chr6.87845121.87845363.–</td><td>Gag</td><td>GAG;GAG_lentiviridae</td><td>.</td><td>Cnbp;ENSMUSP00000109247</td><td></td><td></td></tr>
<tr><td>Mmus38.chr7.104271650.104273401.–</td><td>Gag</td><td>gag;Gag_p24|gag;zf-CCHC_5|GAG;GAG_betaretroviridae|GAG;GAG_lentiviridae</td><td>IAPEz-int;LTR/ERVK</td><td>Trim5;ENSMUSP00000050084</td><td></td><td></td></tr>
<tr><td>Mmus38.chr7.142380732.142380977.–</td><td>Pro</td><td>pro;Asp|AP;AP_pepsins_A1a</td><td>.</td><td>Ctsd;ENSMUSP00000063904</td><td></td><td>0</td></tr>
<tr><td>Mmus38.chr7.143635667.143636467.–</td><td>Pol</td><td>pol;RNase_H</td><td>MERVL-int;LTR/ERVL</td><td>Tnfrsf22;ENSMUSP00000126384</td><td></td><td></td></tr>
<tr><td>Mmus38.chr8.70259617.70259880.+</td><td>Pro</td><td>pro;G-patch</td><td>.</td><td>Sugp2;ENSMUSP00000128029</td><td></td><td></td></tr>
<tr><td>Mmus38.chr12.109590172.109595502.–</td><td>Pro:Gag</td><td>pro;gag-asp_proteas|AP;AP_v_clade|GAG;GAG_v_clade|RT;RT_17_6|RT;RT_crm|RT;RT_del|RT;RT_pyret|RT;RT_reina|RT;RT_v_clade</td><td>(TCC)n;Simple_repeat|(CTCAGC)n;Simple_repeat</td><td>Rtl1;ENSMUSP00000115957</td><td>SINGL</td><td>0</td></tr>
<tr><td>Mmus38.chr14.43930744.43931031.+</td><td>Pol</td><td>pol;IN_DBD_C|INT;INT_betaretroviridae</td><td>IAPEY4_I-int;LTR/ERVK</td><td>Gm8113;ENSMUSP00000125038</td><td></td><td>5402</td></tr>
<tr><td>Mmus38.chr14.69290950.69292893.–</td><td>Env</td><td>ENV;ENV_retroviridae</td><td>RodERV21-int;LTR/ERV1</td><td>Synb;ENSMUSP00000061107</td><td>SINGL</td><td>4431</td></tr>
<tr><td>Mmus38.chr14.73660751.73663234.+</td><td>LINE</td><td>pol;RVT_1</td><td>L1Md_A;LINE/L1</td><td>Gm21750;ENSMUSP00000096471</td><td></td><td></td></tr>
<tr><td>Mmus38.chr16.15317458.15321357.+</td><td>LINE</td><td>pol;RVT_1</td><td>L1Md_T;LINE/L1</td><td>Gm21897;ENSMUSP00000135960</td><td></td><td></td></tr>
<tr><td>Mmus38.chrX.101563477.101563740.+</td><td>Gag</td><td>gag;zf-CCHC_6</td><td>.</td><td>Taf1;ENSMUSP00000098895</td><td></td><td></td></tr>
<tr><td>Mmus38.chrX.94356887.94357219.–</td><td>Gag</td><td>gag;zf-CCHC_6</td><td>(CT)n;Simple_repeat</td><td>Fam90a1b;ENSMUSP00000109536</td><td></td><td></td></tr>
<tr><td>Mmus38.chrY.70293570.70293914.–</td><td>Pol</td><td>pol;rve|INT;INT_epsilonretroviridae|INT;INT_gammaretroviridae</td><td>MuRRS4-int;LTR/ERV1</td><td>Gm29423;ENSMUSP00000140691</td><td></td><td></td></tr>
</table>

Rat ERV-ORFs

<table>
<tr><th>ID</th><th>Domain[</th><th>HMM</th><th>Repbase</th><th>Gene</th></tr>
<tr><td>Rnor50.chr1.222441769.222442107.−</td><td>Pro</td><td>pro;Asp|AP;AP_pepsins_A1a</td><td>.</td><td>Ctsd;ENSRNOP00000027407</td></tr>
<tr><td>Rnor50.chr1.51190516.51190869.+</td><td>Pro</td><td>pro;dUTPase|DUT;DUT_caulimoviruses|DUT;DUT_lentiviridae</td><td>.</td><td>AABR06002285.1;ENSRNOP00000023387</td></tr>
<tr><td>Rnor50.chr1.73779954.73782599.−</td><td>LINE</td><td>pol;RVT_1</td><td>L1_Rat2;LINE/L1</td><td>AABR06003466.1;ENSRNOP00000012225</td></tr>
<tr><td>Rnor50.chr2.132087070.132091020.−</td><td>LINE</td><td>pol;RVT_1</td><td>RNHAL1;LINE/L1</td><td>AABR06015425.1;ENSRNOP00000067570</td></tr>
<tr><td>Rnor50.chr2.221363325.221367224.+</td><td>LINE</td><td>pol;RVT_1</td><td>L1_Rn;LINE/L1</td><td>AABR06019553.1;ENSRNOP00000064593</td></tr>
<tr><td>Rnor50.chr4.185001418.185001660.−</td><td>Gag</td><td>GAG;GAG_lentiviridae</td><td>.</td><td>Cnbp;ENSRNOP00000013884</td></tr>
<tr><td>Rnor50.chr4.221377781.221378269.+</td><td>LINE</td><td>pol;RVT_1</td><td>L1_Rn;LINE/L1</td><td>Mug2;ENSRNOP00000064788</td></tr>
<tr><td>Rnor50.chr6.137174293.137175420.+</td><td>LINE</td><td>pol;RVT_1</td><td>L1_Rat2;LINE/L1</td><td>LOC500712;ENSRNOP00000029694</td></tr>
<tr><td>Rnor50.chr6.142874258.142877095.−</td><td>Gag</td><td>GAG;GAG_v_clade|RT;RT_crm|RT;RT_del|RT;RT_pyret|RT;RT_reina|RT;RT_v_clade</td><td>(TCCTCA)n;Simple_repeat|(ATCTTC)n;Simple_repeat</td><td>Rtl1;ENSRNOP00000066132</td></tr>
<tr><td>Rnor50.chr7.95855614.95857533.+</td><td>Pol</td><td>pol;RVT_1</td><td>L1_Rn;LINE/L1</td><td>Col14a1;ENSRNOP00000063778</td></tr>
<tr><td>Rnor50.chr9.110750764.110751060.−</td><td>Pol</td><td>INT;GIN1</td><td>.</td><td>Gin1;ENSRNOP00000016028</td></tr>
<tr><td>Rnor50.chr9.52775737.52777266.−</td><td>Pol</td><td>pol;IN_DBD_C|pol;Integrase_Zn|pol;RNase_H|pol;rve|INT;INT_alpharetroviridae|INT;INT_betaretroviridae|INT;INT_deltaretroviridae|INT;INT_gammaretroviridae|INT;INT_lentiviridae|INT;INT_spumaretroviridae|RNaseH;RNaseH_betaretroviridae</td><td>RNLTR4d_I;LTR/ERVK</td><td>Ormdl1;ENSRNOP00000061507</td></tr>
<tr><td>Rnor50.chr9.52779885.52780949.−</td><td>Gag</td><td>GAG;GAG_betaretroviridae</td><td>RNLTR4d_I;LTR/ERVK</td><td>Ormdl1;ENSRNOP00000061507</td></tr>
<tr><td>Rnor50.chr10.70117964.70118332.−</td><td>Gag</td><td>gag;Gag_p24|GAG;GAG_betaretroviridae</td><td>MYSERV_Rn-int;LTR/ERVK</td><td>Rn50_10_0702.5;ENSRNOP00000012874</td></tr>
<tr><td>Rnor50.chr12.27165685.27167544.+</td><td>Env</td><td>ENV;ENV_gammaretroviridae|ENV;ENV_retroviridae</td><td>RodERV21-int;LTR/ERV1</td><td>Syna;ENSRNOP00000067387</td></tr>
<tr><td>Rnor50.chr13.116482035.116484032.−</td><td>Pol</td><td>pol;RVT_1</td><td>L1_Rat1;LINE/L1</td><td>Hsd11b1;ENSRNOP00000050581</td></tr>
<tr><td>Rnor50.chr15.54904810.54906750.−</td><td>Env</td><td>ENV;ENV_gammaretroviridae|ENV;ENV_retroviridae</td><td>RodERV21-int;LTR/ERV1</td><td>Synb;ENSRNOG00000021823</td></tr>
<tr><td>Rnor50.chr16.20764085.20764363.−</td><td>Pro</td><td>pro;G-patch</td><td>.</td><td>Sugp2;ENSRNOP00000027457</td></tr>
<tr><td>Rnor50.chr17.46488518.46491379.+</td><td>Pol</td><td>pol;RVT_1</td><td>RNHAL1;LINE/L1|L1_Rn;LINE/L1</td><td>AABR06091708.2;ENSRNOP00000066488</td></tr>
<tr><td>Rnor50.chr17.47415263.47417911.+</td><td>Pol</td><td>pol;RVT_1</td><td>RNHAL1;LINE/L1</td><td>Pou6f2;ENSRNOP00000017713</td></tr>
<tr><td>Rnor50.chr19.32976355.32980209.−</td><td>LINE</td><td>pol;RVT_1</td><td>L1_Rn;LINE/L1</td><td>AABR06097607.1;ENSRNOP00000065391</td></tr>
<tr><td>Rnor50.chr19.47076640.47077344.+</td><td>Pol</td><td>pol;rve|INT;INT_betaretroviridae|INT;INT_deltaretroviridae|INT;INT_gammaretroviridae</td><td>RMER16-int;LTR/ERVK</td><td>AABR06098457.1;ENSRNOP00000038976</td></tr>
</table>

Rabbit ERV-ORFs

ID	Domain	HMM	Repbase	Gene
Ocun2.chr1.182294471.182297680.−	Pol	pol;RVT_1	Sat−1_TSy;Satellite|L1A2_OC;LINE/L1	NA;ENSOCUG00000029631
Ocun2.chr1.192706896.192709235.+	LINE	pol;RVT_1	L1A_Oc;LINE/L1|Sat−1_TSy;Satellite|L1A2_OC;LINE/L1	NA;ENSOCUG00000029694
Ocun2.chr2.116246603.116247574.+	Pro	AP;AP_DTG_ILG_template|AP;AP_saspase	.	ASPRV1;ENSOCUG00000015830
Ocun2.chr21.12630468.12634019.−	Pol	pol;IN_DBD_C|pol;rve|INT;INT_alpharetroviridae|INT;INT_betaretroviridae|INT;INT_deltaretroviridae|INT;INT_gammaretroviridae|INT;INT_lentiviridae|INT;INT_spumaretroviridae|RNaseH;RNaseH_betaretroviridae	ERV2−2_OC−I;LTR/ERVK	NA;ENSOCUG00000029571
Ocun2.chr7.158233001.158236747.+	LINE	pol;RVT_1	L1A_Oc;LINE/L1|Sat−1_TSy;Satellite	NA;ENSOCUG00000029715
Ocun2.chr7.77715477.77718938.−	LINE	pol;RVT_1	Sat−1_TSy;Satellite|L1A_Oc;LINE/L1	NA;ENSOCUG00000029595
Ocun2.chr9.7824836.7825078.+	Gag	GAG;GAG_lentiviridae	.	CNBP;ENSOCUG00000008696
Ocun2.chr10.34278491.34279759.−	Pro	AP;AP_v_clade	.	PEG10;ENSOCUG00000027535
Ocun2.chr10.34279045.34279962.−	Gag	GAG;GAG_v_clade	.	PEG10;ENSOCUG00000027535
Ocun2.chr11.22459697.22460035.−	Pol	INT;GIN1	.	GIN1;ENSOCUG00000025993
Ocun2.chr12.5569750.5572134.−	Pol	pol;IN_DBD_C|pol;Integrase_Zn|pol;RNase_H|pol;rve|pol;RVT_1|pol;RVT_thumb|INT;INT_a_clade|INT;INT_alpharetroviridae|INT;INT_b_clade|INT;INT_betaretroviridae|INT;INT_deltaretroviridae|INT;INT_gammaretroviridae|INT;INT_lentiviridae|INT;INT_spumaretroviridae|RNaseH;RNaseH_betaretroviridae|RT;RT_alpharetroviridae|RT;RT_betaretroviridae|RT;RT_deltaretroviridae|RT;RT_gammaretroviridae|RT;RT_lentiviridae	ERVH_OC_I−int;LTR/ERVK	NA;ENSOCUG00000029713
Ocun2.chr12.5571980.5572339.−	Pol	RT;RT_alpharetroviridae|RT;RT_betaretroviridae|RT;RT_epsilonretroviridae|RT;RT_gammaretroviridae|RT;RT_lentiviridae	ERVH_OC_I−int;LTR/ERVK	NA;ENSOCUG00000029713
Ocun2.chr12.99949107.99950864.+	Env	env;TLV_coat|ENV;ENV_deltaretroviridae|ENV;ENV_D−type_betaretroviridae|ENV;ENV_gammaretroviridae|ENV;ENV_retroviridae	MacERV4_int−int;LTR/ERVK|KORV_I−int;LTR/ERV1	ORY1;ENSOCUG00000029640
Ocun2.chr13.102787390.102790377.−	LINE	pol;RVT_1	Sat−1_TSy;Satellite|L1A_Oc;LINE/L1	NA;ENSOCUG00000029537
Ocun2.chr13.24336471.24339437.−	LINE	pol;RVT_1	Sat−1_TSy;Satellite|L1A_Oc;LINE/L1	NA;ENSOCUG00000029477
Ocun2.chr18.22458305.22462174.−	LINE	pol;RVT_1	L1C_OC;LINE/L1|Sat−1_TSy;Satellite|L1A_Oc;LINE/L1	NA;ENSOCUG00000029155
Ocun2.chrX.16160870.16163458.+	Pol	pol;IN_DBD_C|pol;Integrase_Zn|pol;RNase_H|pol;rve|pol;RVT_1|pol;RVT_thumb|INT;INT_a_clade|INT;INT_alpharetroviridae|INT;INT_betaretroviridae|INT;INT_deltaretroviridae|INT;INT_gammaretroviridae|INT;INT_lentiviridae|INT;INT_spumaretroviridae|RNaseH;RNaseH_betaretroviridae|RT;RT_17_6|RT;RT_alpharetroviridae|RT;RT_badnavirus|RT;RT_betaretroviridae|RT;RT_deltaretroviridae|RT;RT_epsilonretroviridae|RT;RT_gammaretroviridae|RT;RT_lentiviridae|RT;RT_spumaretroviridae	ERVH_OC_I−int;LTR/ERVK	NA;ENSOCUG00000029748
Ocun2.chrX.49877023.49877274.+	Gag	gag;zf−CCHC_6	.	NA;ENSOCUG00000015656
Ocun2.chrX.62570394.62572061.+	Env	env;TLV_coat|ENV;ENV_deltaretroviridae|ENV;ENV_D−type_betaretroviridae|ENV;ENV_retroviridae	MacERV4_int−int;LTR/ERVK|KORV_I−int;LTR/ERV1	NA;ENSOCUG00000029381

Dog ERV-ORFs

<table>
<tr><th>ID</th><th>Domain</th><th>HMM</th><th>Repbase</th><th>Gene</th></tr>
<tr><td>Cfam31.chr2.10125398.10125826.-</td><td>Pol</td><td>INT;INT_epsilonretroviridae|INT;INT_gammaretroviridae</td><td>CfERV1-int;LTR/ERV1</td><td>NA;ENSCAFP00000037385</td></tr>
<tr><td>Cfam31.chr2.10125562.10125852.-</td><td>Pol</td><td>RT;RT_epsilonretroviridae|RT;RT_gammaretroviridae</td><td>CfERV1-int;LTR/ERV1</td><td>NA;ENSCAFP00000037385</td></tr>
<tr><td>Cfam31.chr2.10125762.10126721.-</td><td>Pro</td><td>AP;AP_gammaretroviridae|AP;AP_retropepsins|RT;RT_epsilonretroviridae|RT;RT_gammaretroviridae</td><td>CfERV1-int;LTR/ERV1</td><td>NA;ENSCAFP00000037385</td></tr>
<tr><td>Cfam31.chr3.8115754.8116047.+</td><td>Pol</td><td>INT;GIN1</td><td>.</td><td>GIN1;ENSCAFP00000011199</td></tr>
<tr><td>Cfam31.chr3.82194695.82195876.-</td><td>Env</td><td>env;TLV_coat|ENV;ENV_D-type_betaretroviridae|ENV;ENV_gammaretroviridae|ENV;ENV_retroviridae</td><td>CfERVF1-int;LTR/ERV1</td><td>NA;ENSCAFP00000039733</td></tr>
<tr><td>Cfam31.chr3.82196099.82198096.-</td><td>Pol</td><td>pol;RNase_H|pol;rve|INT;INT_b_clade|INT;INT_betaretroviridae|INT;INT_csrn1|INT;INT_deltaretroviridae|INT;INT_epsilonretroviridae|INT;INT_gammaretroviridae|INT;INT_gmr1|INT;INT_lentiviridae|INT;INT_spumaretroviridae|INT;INT_tat|RNaseH;RNaseH_epsilonretroviridae|RNaseH;RNaseH_gammaretroviridae|RNaseH;RNaseH_spumaretroviridae</td><td>CfERVF1-int;LTR/ERV1</td><td>NA;ENSCAFP00000039733</td></tr>
<tr><td>Cfam31.chr3.83844409.83845833.+</td><td>Env</td><td>ENV;ENV_retroviridae</td><td>CarERV3-int;LTR?ERV1</td><td>CAR1;NA</td></tr>
<tr><td>Cfam31.chr5.963770.964429.+</td><td>LINE</td><td>pol;RVT_1</td><td>L1_Canis1;LINE/L1</td><td>NA;ENSCAFP00000042660</td></tr>
<tr><td>Cfam31.chr8.69103085.69106390.-</td><td>Pro</td><td>AP;AP_v_clade|GAG;GAG_v_clade|RT;RT_17_6|RT;RT_crm|RT;RT_del|RT;RT_gypsy|RT;RT_maggy|RT;RT_pyggy|RT;RT_pyret|RT;RT_reina|RT;RT_v_clade</td><td>(CTC)n;Simple_repeat</td><td>RTL1;ENSCAFP00000026463</td></tr>
<tr><td>Cfam31.chr8.8320179.8322245.-</td><td>LINE</td><td>pol;RVT_1</td><td>L1_Cf;LINE/L1</td><td>NA;ENSCAFP00000033727</td></tr>
<tr><td>Cfam31.chr10.3285576.3286484.+</td><td>Env</td><td>env;TLV_coat|ENV;ENV_D-type_betaretroviridae|ENV;ENV_gammaretroviridae|ENV;ENV_retroviridae</td><td>CfERV1-int;LTR/ERV1</td><td>NA;ENSCAFP00000037516</td></tr>
<tr><td>Cfam31.chr10.68586978.68587886.-</td><td>Pro</td><td>AP;AP_DTG_ILG_template|AP;AP_saspase</td><td>(CCGTCC)n;Simple_repeat|(GCAGGT)n;Simple_repeat</td><td>ASPRV1;ENSCAFP0000000492[illegible]</td></tr>
<tr><td>Cfam31.chr12.32255437.32256183.+</td><td>Env</td><td>env;GP41|ENV;ENV_B-type_betaretroviridae</td><td>(T)n;Simple_repeat</td><td>NA;ENSCAFP00000042124</td></tr>
<tr><td>Cfam31.chr12.49629127.49631124.-</td><td>Env</td><td>env;GP41|ENV;ENV_B-type_betaretroviridae|ENV;ENV_retroviridae</td><td>.</td><td>NA;ENSCAFP00000039835</td></tr>
<tr><td>Cfam31.chr14.20127798.20128877.+</td><td>Gag</td><td>GAG;GAG_v_clade</td><td>(CCT)n;Simple_repeat</td><td>PEG10;ENSCAFP00000041426</td></tr>
<tr><td>Cfam31.chr14.20128379.20129443.+</td><td>Pro</td><td>AP;AP_v_clade</td><td>.</td><td>PEG10;ENSCAFP00000041426</td></tr>
<tr><td>Cfam31.chr16.54089590.54089898.-</td><td>Gag</td><td>gag;zf-CCHC_6</td><td>.</td><td>NA;ENSCAFP00000034730</td></tr>
<tr><td>Cfam31.chr18.46013222.46013488.-</td><td>Pro</td><td>pro;Asp|AP;AP_pepsins_A1a</td><td>.</td><td>NA;ENSCAFP00000014791</td></tr>
<tr><td>Cfam31.chr20.2926592.2926834.+</td><td>Gag</td><td>GAG;GAG_lentiviridae</td><td>.</td><td>CNBP;ENSCAFP00000006421</td></tr>
<tr><td>Cfam31.chr20.44128813.44129130.+</td><td>Pro</td><td>pro;G-patch</td><td>.</td><td>SUGP2;ENSCAFP00000021327</td></tr>
<tr><td>Cfam31.chr26.28604208.28605758.+</td><td>Gag</td><td>gag;Gag_p30</td><td>MER66-int;LTR/ERV1|(CCAGCC)n;Simple_repeat</td><td>NA;ENSCAFP00000037400</td></tr>
<tr><td>Cfam31.chr35.24271063.24272484.+</td><td>Env</td><td>env;TLV_coat|ENV;ENV_gammaretroviridae|ENV;ENV_retroviridae</td><td>MER66-int;LTR/ERV1</td><td>NA;ENSCAFP00000038239</td></tr>
<tr><td>Cfam31.chr35.24993046.24993933.-</td><td>Pol</td><td>pol;rve|INT;INT_b_clade|INT;INT_deltaretroviridae|INT;INT_epsilonretroviridae|INT;INT_gammaretroviridae|INT;INT_gmr1|INT;INT_lentiviridae|INT;INT_osvaldo|INT;INT_spumaretroviridae</td><td>CfERV1-int;LTR/ERV1</td><td>NA;ENSCAFP00000042892</td></tr>
<tr><td>Cfam31.chrX.55720071.55720400.+</td><td>Gag</td><td>gag;zf-CCHC_6</td><td>.</td><td>NA;ENSCAFP00000025204</td></tr>
</table>

Cat ERV-ORFs

<table>
<tr><th>ID</th><th>Domain</th><th>HMM</th><th>Repbase</th><th>Gene</th></tr>
<tr><td>Fcat62.chrA1.163926192.163926512.-</td><td>Pol</td><td>Pol;RVT_1</td><td>.</td><td>GIN1;ENSFCAP00000025585</td></tr>
<tr><td>Fcat62.chrA2.98418392.98419471.+</td><td>Gag</td><td>GAG;GAG_v_clade</td><td>(CCT)n;Simple_repeat</td><td>PEG10;ENSFCAP00000024585</td></tr>
<tr><td>Fcat62.chrA2.13751269.13751583.-</td><td>Pro</td><td>pro;G-patch</td><td>.</td><td>SUGP2;ENSFCAP00000009055</td></tr>
<tr><td>Fcat62.chrB1.138206424.138207278.-</td><td>LINE</td><td>pol;RVT_1</td><td>L1-2_Fc;LINE/L1</td><td>NA;ENSFCAP00000017244</td></tr>
<tr><td>Fcat62.chrB1.183859927.183861351.+</td><td>Env</td><td>ENV;ENV_gammaretroviridae</td><td>CarERV3-int;LTR?ERV1</td><td>CAR1; ENSFCAG00000028607</td></tr>
<tr><td>Fcat62.chrB2.3491740.3493038.-</td><td>Env</td><td>ENV;ENV_gammaretroviridae|ENV;ENV_retroviridae</td><td>CarERV2-int;LTR/ERV1</td><td>NA;ENSFCAP00000024081</td></tr>
<tr><td>Fcat62.chrA3.89267109.89268092.–</td><td>Pro</td><td>AP;AP_DTG_ILG_template|AP;AP_saspase</td><td>.</td><td>ASPRV1;ENSFCAP00000017297</td></tr>
<tr><td>Fcat62.chrB4.47615806.47616552.+</td><td>Gag</td><td>GAG;GAG_gammaretroviridae</td><td>ERV1-3_FCa-I;LTR/ERV1</td><td>NA;ENSFCAP00000025368</td></tr>
<tr><td>Fcat62.chrB4.47616233.47616805.+</td><td>Gag</td><td>GAG;GAG_gammaretroviridae</td><td>ERV1-3_FCa-I;LTR/ERV1</td><td>NA;ENSFCAP00000025368</td></tr>
<tr><td>Fcat62.chrB4.47618106.47618432.+</td><td>Gag</td><td>gag;Gag_p30|GAG;GAG_gammaretroviridae</td><td>ERV1-3_FCa-I;LTR/ERV1</td><td>NA;ENSFCAP00000025368</td></tr>
<tr><td>Fcat62.chrB4.47618264.47618530.+</td><td>Gag</td><td>gag;Gag_p30|GAG;GAG_gammaretroviridae</td><td>ERV1-3_FCa-I;LTR/ERV1</td><td>NA;ENSFCAP00000025368</td></tr>
<tr><td>Fcat62.chrB4.47618436.47619767.+</td><td>Gag</td><td>gag;Gag_p30|pol;rve|GAG;GAG_gammaretroviridae|INT;INT_b_clade|INT;INT_epsilonretroviridae|INT;INT_gammaretroviridae|INT;INT_gmr1|INT;INT_spumaretroviridae</td><td>ERV1-3_FCa-I;LTR/ERV1</td><td>NA;ENSFCAP00000025368</td></tr>
<tr><td>Fcat62.chrB4.47619368.47620531.+</td><td>Env</td><td>env;TLV_coat</td><td>ERV1-3_FCa-I;LTR/ERV1</td><td>NA;ENSFCAP00000025368</td></tr>
<tr><td>Fcat62.chrC1.93051406.93051657.+</td><td>Pro</td><td>AP;AP_pepsins_A1a</td><td>.</td><td>NA;ENSFCAP00000019684</td></tr>
<tr><td>Fcat62.chrD1.104121318.104122013.-</td><td>Gag</td><td>gag;Gag_p30|GAG;GAG_gammaretroviridae</td><td>ERV1-3_FCa-I;LTR/ERV1</td><td>SLC43A1;ENSFCAP00000010539</td></tr>
<tr><td>Fcat62.chrD1.115549054.115549392.+</td><td>Pro</td><td>pro;Asp|AP;AP_pepsins_A1a</td><td>.</td><td>NA;ENSFCAP00000007246</td></tr>
<tr><td>Fcat62.chrE2.7357550.7357795.+</td><td>Pro</td><td>AP;AP_pepsins_A1a</td><td>.</td><td>NAPSA;ENSFCAP00000000296</td></tr>
<tr><td>Fcat62.chrX.55076453.55076968.+</td><td>Pro</td><td>pro;dUTPase|DUT;DUT_caulimoviruses</td><td>(CTCTG)n;Simple_repeat</td><td>NA;ENSFCAP00000017174</td></tr>
<tr><td>Fcat62.chrX.59136384.59136827.+</td><td>Gag</td><td>gag;zf-CCHC_6</td><td>SINEC_Fc2;SINE/tRNA</td><td>NA;ENSFCAP00000013737</td></tr>
</table>

Pig ERV-ORFs

ID	Domain	HMM	Repbase	Gene
Sscr102.chr1.147292572.147293012.+	Pol	INT;INT_epsilonretroviridae|INT;INT_gammaretroviridae	KORV_I-int;LTR/ERV1	NA;ENSSSCP00000021333
Sscr102.chr1.147292864.147294852.+	Env	env;TLV_coat|ENV;ENV_deltaretroviridae|ENV;ENV_D-type_betaretroviridae|ENV;ENV_gammaretroviridae|ENV;ENV_retroviridae	KORV_I-int;LTR/ERV1	NA;ENSSSCP00000021333
Sscr102.chr2.112579816.112580115.-	Pol	INT;GIN1	.	GIN1;ENSSSCP00000026307
Sscr102.chr2.58660862.58661152.+	Pro	pro;G-patch	.	SUGP2;ENSSSCP00000022837
Sscr102.chr2.77165435.77165860.+	Pol	INT;INT_epsilonretroviridae|INT;INT_gammaretroviridae	KORV_I-int;LTR/ERV1	NA;ENSSSCP00000023068
Sscr102.chr2.77165712.77166596.+	Env	env;TLV_coat	KORV_I-int;LTR/ERV1	NA;ENSSSCP00000023068
Sscr102.chr7.132097261.132100221.-	Pro	pro;gag-asp_proteas|AP;AP_v_clade|RT;RT_17_6|RT;RT_crm|RT;RT_del|RT;RT_gypsy|RT;RT_maggy|RT;RT_pyggy|RT;RT_v_clade	(TCC)n;Simple_repeat	RTL1;ENSSSCP00000019531
Sscr102.chr7.42279971.42280228.+	Pro	AP;AP_pepsins_A1a	.	PGC;ENSSSCP00000001761
Sscr102.chr15.128593496.128594404.-	Env	env;TLV_coat	KORV_I-int;LTR/ERV1|ERV1-2_SSc-I;LTR/ERV1	NA;ENSSSCP00000023429
Sscr102.chr15.128594256.128594687.-	Pol	INT;INT_epsilonretroviridae|INT;INT_gammaretroviridae	ERV1-2_SSc-I;LTR/ERV1	NA;ENSSSCP00000023429
Sscr102.chr16.10274963.10276945.+	Pol	pol;Integrase_Zn|pol;RNase_H|pol;RVT_1|pol;RVT_thumb|INT;INT_betaretroviridae|INT;INT_deltaretroviridae|INT;INT_gammaretroviridae|RNaseH;RNaseH_betaretroviridae|RT;RT_17_6|RT;RT_alpharetroviridae|RT;RT_badnavirus|RT;RT_betaretroviridae|RT;RT_deltaretroviridae|RT;RT_gammaretroviridae|RT;RT_gmr1|RT;RT_lentiviridae|RT;RT_spumaretroviridae	ERV2-2-EC_I-int;LTR/ERVK	NA;ENSSSCP00000027525
Sscr102.chrX.95525871.95526314.+	Pol	INT;INT_epsilonretroviridae|INT;INT_gammaretroviridae	KORV_I-int;LTR/ERV1	NA;ENSSSCP00000025206
Sscr102.chrX.95526763.95528154.+	Env	env;TLV_coat|ENV;ENV_deltaretroviridae|ENV;ENV_D-type_betaretroviridae|ENV;ENV_gammaretroviridae|ENV;ENV_retroviridae	KORV_I-int;LTR/ERV1	NA;ENSSSCP00000025206

Cow ERV-ORFs

<table>
<tr><th>ID</th><th>Domain</th><th>HMM</th><th>Repbase</th><th>Gene</th></tr>
<tr><td>BtauUMD31.chr2.89843949.89844566.+</td><td>LINE</td><td>pol;RVT_1</td><td>BovB;LINE/RTE-BovB|ART2A;SINE/RTE-BovB</td><td>AOX2;ENSBTAP00000038143</td></tr>
<tr><td>BtauUMD31.chr3.54421563.54422549.+</td><td>LINE</td><td>pol;RVT_1</td><td>BovB;LINE/RTE-BovB|ART2A;SINE/RTE-BovB</td><td>NA;ENSBTAP00000048119</td></tr>
<tr><td>BtauUMD31.chr3.54951197.54951937.+</td><td>LINE</td><td>pol;RVT_1</td><td>BovB;LINE/RTE-BovB|ART2A;SINE/RTE-BovB</td><td>NA;ENSBTAP00000019081</td></tr>
<tr><td>BtauUMD31.chr4.18061526.18062050.–</td><td>LINE</td><td>pol;RVT_1</td><td>BovB;LINE/RTE-BovB</td><td>NA;ENSBTAP00000055238</td></tr>
<tr><td>BtauUMD31.chr5.29427372.29428259.+</td><td>LINE</td><td>pol;RVT_1</td><td>L1_BT;LINE/L1</td><td>NA;ENSBTAP00000013453</td></tr>
<tr><td>BtauUMD31.chr5.85305102.85305743.+</td><td>LINE</td><td>pol;RVT_1</td><td>SINE2-1_BT;SINE/tRNA|L1_BT;LINE/L1</td><td>CASC1;ENSBTAP00000050480</td></tr>
<tr><td>BtauUMD31.chr6.14296424.14296819.–</td><td>LINE</td><td>pol;RVT_1</td><td>BovB;LINE/RTE-BovB</td><td>ALPK1;ENSBTAP00000013457</td></tr>
<tr><td>BtauUMD31.chr7.4139270.4139530.+</td><td>Pro:Pol</td><td>pro;G-patch</td><td>.</td><td>SUGP2;ENSBTAP00000017672</td></tr>
<tr><td>BtauUMD31.chr7.64723687.64725831.+</td><td>Env</td><td>env;GP41|ENV;ENV_B type_bertaretroviridae</td><td>ERV2-1-I_BT;LTR/ERVK|BTLTR1F;LTR/ERVK</td><td>Fematrin-1 (BERV-K1 env)</td></tr>
<tr><td>BtauUMD31.chr7.104530493.104530870.–</td><td>Pol</td><td>INT;GIN1</td><td>(AAATA)n;Simple_repeat</td><td>GIN1;ENSBTAP00000050552</td></tr>
<tr><td>BtauUMD31.chr10.76487509.76487952.+</td><td>LINE</td><td>pol;RVT_1</td><td>ART2A;SINE/RTE-BovB|BovB;LINE/RTE-BovB</td><td>SYNE2;ENSBTAP00000029285</td></tr>
<tr><td>BtauUMD31.chr12.18173795.18174319.–</td><td>LINE</td><td>pol;RVT_1</td><td>BovB;LINE/RTE-BovB</td><td>NA;ENSBTAP00000004630</td></tr>
<tr><td>BtauUMD31.chr12.79021757.79022221.+</td><td>LINE</td><td>pol;RVT_1</td><td>BovB;LINE/RTE-BovB</td><td>IPO5;ENSBTAP00000043010</td></tr>
<tr><td>BtauUMD31.chr13.9834956.9835924.+</td><td>LINE</td><td>pol;RVT_1</td><td>BovB;LINE/RTE-BovB|ART2A;SINE/RTE-BovB</td><td>MACROD2;ENSBTAP00000045773</td></tr>
<tr><td>BtauUMD31.chr13.78407524.78408978.–</td><td>Env</td><td>env;TLV_coat|ENV;ENV_retroviridae</td><td>ERV1N-3_SSc-I;LTR/ERV1</td><td>RUM1;ENSBTAP00000062096</td></tr>
<tr><td>BtauUMD31.chr14.26441553.26442059.+</td><td>LINE</td><td>pol;RVT_1</td><td>BovB;LINE/RTE-BovB</td><td>SDCBP;ENSBTAP00000026526</td></tr>
<tr><td>BtauUMD31.chr14.59410045.59410341.+</td><td>LINE</td><td>pol;RVT_1</td><td>BovB;LINE/RTE-BovB</td><td>ANGPT1;ENSBTAP00000018675</td></tr>
<tr><td>BtauUMD31.chr17.54174017.54174778.–</td><td>LINE</td><td>pol;RVT_1</td><td>ART2A;SINE/RTE-BovB|BovB;LINE/RTE-BovB</td><td>DNAH10;ENSBTAP00000029662</td></tr>
<tr><td>BtauUMD31.chr18.29556085.29558388.–</td><td>LINE</td><td>pol;RVT_1</td><td>L1_BT;LINE/L1</td><td>CDH8;ENSBTAP00000030891</td></tr>
<tr><td>BtauUMD31.chr19.40030158.40030817.–</td><td>LINE</td><td>pol;RVT_1</td><td>ART2A;SINE/RTE-BovB|BovB;LINE/RTE-BovB</td><td>CWC25;ENSBTAP00000040794</td></tr>
<tr><td>BtauUMD31.chr21.55339309.55339986.+</td><td>LINE</td><td>pol;RVT_1</td><td>BovB;LINE/RTE-BovB|ART2A;SINE/RTE-BovB</td><td>NA;ENSBTAP00000040927</td></tr>
<tr><td>BtauUMD31.chr21.67427517.67431587.–</td><td>Pro:Pol</td><td>pro;gag-asp_proteas|GAG;GAG_v_clade|RT;RT_17_6|RT;RT_crm|RT;RT_del|RT;RT_gypsy|RT;RT_maggy| RT;RT_pyggy|RT;RT_v_clade</td><td>.</td><td>RTL1;ENSBTAP00000055386</td></tr>
<tr><td>BtauUMD31.chr22.11528351.11529376.+</td><td>LINE</td><td>pol;RVT_1</td><td>BovB;LINE/RTE-BovB|BTLTR1;LTR/ERVK</td><td>DLEC1;ENSBTAP00000011298</td></tr>
<tr><td>BtauUMD31.chr22.55015756.55017120.–</td><td>LINE</td><td>pol;RVT_1</td><td>ART2A;SINE/RTE-BovB|BovB;LINE/RTE-BovB|BTLTR1;LTR/ERVK</td><td>ATP2B2;ENSBTAP00000001723</td></tr>
<tr><td>BtauUMD31.chr24.12817236.12819377.+</td><td>Env</td><td>env;GP41|ENV;ENV_B type_bertaretroviridae</td><td>ERV2-1-I_BT;LTR/ERVK|ERV2-1-LTR_BT;LTR/ERVK</td><td>BERV-K2 env</td></tr>
<tr><td>BtauUMD31.chr25.19468570.19469076.+</td><td>LINE</td><td>pol;RVT_1</td><td>BovB;LINE/RTE-BovB</td><td>NA;ENSBTAP00000008832</td></tr>
<tr><td>BtauUMD31.chr28.12921485.12921745.+</td><td>LINE</td><td>pol;RVT_1</td><td>BovB;LINE/RTE-BovB</td><td>ZNF33B;ENSBTAP00000024349</td></tr>
<tr><td>BtauUMD31.chrX.84544746.84545081.–</td><td>Gag</td><td>gag;zf-CCHC_6</td><td>.</td><td>NA;ENSBTAP00000053806</td></tr>
</table>

Platypus ERV-ORFs

<table>
<tr><th>ID</th><th>Domain (HMM)</th><th>Repbase</th><th>Gene</th></tr>
<tr><td>Oana5.chrX1.32227928.32228290.+</td><td>pol;RVT_1</td><td>Mon1d;SINE/MIR|L2_Plat1m;LINE/L2</td><td>FGD5;ENSOANP00000017248</td></tr>
</table>

Table S2 Species and Genome assembly used in this study.

Genome ID: Species name in the gEVE database (http://geve.med.u-tokai.ac.jp)

Species	Nomenclature	Genome Database	Genome ID
Human	Homo sapiens	GRCh38, Dec 2013	Hsap38
Chimpanzee	Pan troglodytes	CSAC 2.1.4/panTro4, Feb 2011	Ptro214
Gorilla	Gorilla gorilla gorilla	gorGor3.1/gorGo3, May 2011	Ggor31
Orangutan	Pongo pygmaeus abelii	PPYG2, Sep 2007	Pabe2
Baboon	Papio anubis	Panu_2.0, Jun 2012	Panu2
Macaque	Macaca mulatta	MMUL 1.0, Feb 2006	Mmul1
Marmoset	Callithrix jacchus	C_jacchus3.2.1, Jan 2010	Cjac321
Mouse	Mus musculus	GRCm38.p1, Jan 2012	Mmus38
Rat	Rattus norvegicus	Rnor_5.0, Mar 2012	Rnor50
Rabbit	Oryctolagus cuniculus	oryCun2, Nov 2009	Ocun2
Cow	Bos taurus UMD3.1	UMD3.1, Dec 2009	BtauUMD31
Cow	Bos taurus 4.6.1	Btau_4.6.1 Nov 2011	Btau461
Dog	Canis lupus familiaris	CanFam3.1, Sep 2011	Cfam31
Cat	Felis catus	Felis_catus_6.2, Sep 2011	Fcat62
Horse	Equus caballus	Equ Cab 2, Sep 2007	Ecab2
Sheep	Ovis aries	Oar_v3.1, 2012/09/24	Oari31
Pig	Sus scrofa	Sscrofa10.2, Aug 2011	Sscr102
Goat	Capra hircus	CHIR_1.0, Jan 2013	Chir1
Opossum	Monodelphis domestica	monDom5, Oct 2006	Mdom5
Platypus	Ornithorhynchus anatinus	OANA5, Dec 2005	Oana5

Table S3 Accession numbers of runs used in myoblast differentiation analysis.

A) Human, **B)** Mouse. NOTE: We did not use a mouse replicate SRA data for day3 because it was broken.

A Human (SRP033135)

Run ID	BioSample	Sample name	Experiment	Instrument	Type in this stuc	Library protocol	Label
SRR1033282	SAMN02413273	GSM1269332	SRX379972	Illumina HiSeq 2500	D0	Bulk RNA-Seq (TruSeq)	Bulk RNA-Seq_0 hrs
SRR1033283	SAMN02413261	GSM1269333	SRX379973	Illumina HiSeq 2500	D0	Bulk RNA-Seq (TruSeq)	Bulk RNA-Seq_0 hrs
SRR1033284	SAMN02413275	GSM1269334	SRX379974	Illumina HiSeq 2500	D0	Bulk RNA-Seq (TruSeq)	Bulk RNA-Seq_0 hrs
SRR1033285	SAMN02413262	GSM1269335	SRX379975	Illumina HiSeq 2500	D1	Bulk RNA-Seq (TruSeq)	Bulk RNA-Seq_24 hrs
SRR1033286	SAMN02413276	GSM1269336	SRX379976	Illumina HiSeq 2500	D1	Bulk RNA-Seq (TruSeq)	Bulk RNA-Seq_24 hrs
SRR1033287	SAMN02413264	GSM1269337	SRX379977	Illumina HiSeq 2500	D1	Bulk RNA-Seq (TruSeq)	Bulk RNA-Seq_24 hrs
SRR1033288	SAMN02413277	GSM1269338	SRX379978	Illumina HiSeq 2500	D2	Bulk RNA-Seq (TruSeq)	Bulk RNA-Seq_48 hrs
SRR1033289	SAMN02413263	GSM1269339	SRX379979	Illumina HiSeq 2500	D2	Bulk RNA-Seq (TruSeq)	Bulk RNA-Seq_48 hrs
SRR1033290	SAMN02413278	GSM1269340	SRX379980	Illumina HiSeq 2500	D2	Bulk RNA-Seq (TruSeq)	Bulk RNA-Seq_48 hrs
SRR1033291	SAMN02413265	GSM1269341	SRX379981	Illumina HiSeq 2500	D3	Bulk RNA-Seq (TruSeq)	Bulk RNA-Seq_72 hrs
SRR1033292	SAMN02413279	GSM1269342	SRX379982	Illumina HiSeq 2500	D3	Bulk RNA-Seq (TruSeq)	Bulk RNA-Seq_72 hrs
SRR1033293	SAMN02413266	GSM1269343	SRX379983	Illumina HiSeq 2500	D3	Bulk RNA-Seq (TruSeq)	Bulk RNA-Seq_72 hrs

B Mouse (SRP036149)

Run ID	BioSample	Sample name	Experiment	Instrument	Type in this stuc	LibrarySelection	Label
SRR1156540	SAMN02615744	day0_rep1	SRX460743	Illumina HiSeq 2500	D0	PolyA	day0_rep1
SRR1156548	SAMN02615744	day0_rep1	SRX460743	Illumina HiSeq 2500	D0	PolyA	day0_rep1
SRR1156614	SAMN02615745	day0_rep2	SRX460803	Illumina HiSeq 2500	D0	PolyA	day0_rep2
SRR1156621	SAMN02615745	day0_rep2	SRX460803	Illumina HiSeq 2500	D0	PolyA	day0_rep2
SRR1156654	SAMN02615746	day0_rep3	SRX460855	Illumina HiSeq 2500	D0	PolyA	day0_rep3
SRR1156816	SAMN02615746	day0_rep3	SRX460855	Illumina HiSeq 2500	D0	PolyA	day0_rep3
SRR1156931	SAMN02615747	day3_rep1	SRX461126	Illumina HiSeq 2500	D3	PolyA	day3_rep1
SRR1156937	SAMN02615747	day3_rep1	SRX461126	Illumina HiSeq 2500	D3	PolyA	day3_rep1
SRR1156940	SAMN02615748	day3_rep2	SRX461143	Illumina HiSeq 2500	D3	PolyA	day3_rep2
SRR1156941	SAMN02615748	day3_rep2	SRX461143	Illumina HiSeq 2500	D3	PolyA	day3_rep2
SRR1156943	SAMN02615749	day3_rep3	SRX461144	Illumina HiSeq 2500	D3	PolyA	day3_rep3
SRR1156944	SAMN02615750	day6_rep1	SRX461145	Illumina HiSeq 2500	D6	PolyA	day6_rep1
SRR1156945	SAMN02615750	day6_rep1	SRX461145	Illumina HiSeq 2500	D6	PolyA	day6_rep1
SRR1156946	SAMN02615751	day6_rep2	SRX461146	Illumina HiSeq 2500	D6	PolyA	day6_rep2
SRR1156947	SAMN02615751	day6_rep2	SRX461146	Illumina HiSeq 2500	D6	PolyA	day6_rep2
SRR1156948	SAMN02615752	day6_rep3	SRX461147	Illumina HiSeq 2500	D6	PolyA	day6_rep3
SRR1156949	SAMN02615752	day6_rep3	SRX461147	Illumina HiSeq 2500	D6	PolyA	day6_rep3
